# Effects of the Temperature–Humidity Index on Milk Production Traits and Gene–Environment Interactions in Chinese Holstein Cows

**DOI:** 10.3390/ani16152379

**Published:** 2026-08-03

**Authors:** Kangli Ou, Kun Zheng, Jun Teng, Yan Li, Qin Zhang, Chao Ning, Dan Wang

**Affiliations:** 1 College of Animal Science & Technology, Shandong Agricultural University, Tai’an 271017, China; m18937879659@163.com (K.O.); 13884906372@163.com (K.Z.); tengjun0520@163.com (J.T.); qzhang@sdau.edu.cn (Q.Z.); 2 China Association of Agricultural Science Societies, Beijing 100125, China; liyan@moahr.com

**Keywords:** heat stress, temperature-humidity index, Chinese Holstein cattle, gene-environment interaction, GWAS

## Abstract

Heat stress limits dairy production. The temperature-humidity index (THI), combining temperature and relative humidity, is widely used to assess heat stress. However, in Chinese Holstein cattle, the phenotypic responses of milk traits and genotype-environment interaction mechanisms under different THI conditions are understudied. Based on 63,334 records from 7240 cows (milk yield, fat percentage, protein percentage), matched with meteorological data and 113,297 SNPs, we employed a random-effects GWAS to examine SNP effects across a continuous THI gradient, comparing results with conventional, temperature-, and humidity-interaction GWAS. As THI increased, all traits declined with distinct patterns. Random regression GWAS identified 149 significant SNP × THI interactions (5 for MY, 86 for FP, 58 for PP), distributed across BTA5, BTA6, BTA14, and BTA20. Candidate gene annotation identified 52 candidate genes near significant SNPs, of which 50 core candidate genes were supported in both temperature and humidity GWAS. The most robustly supported core candidate genes include *DGAT1*, *CPSF1*, *ABCG2*, *MGST1*, *VPS28*, *PPP1R16A*, *ZNF250*, *GRID2*, *KCNC2*, and *LOC787350*—of which *DGAT1*, *ABCG2*, and *MGST1* have been functionally validated in milk production traits, whereas others represent novel candidates requiring further investigation. Temperature and THI-GWAS showed high consistency, while humidity-GWAS detected both overlapping and specific signals. Incorporating THI as a continuous environmental gradient identifies environment-dependent regulatory signals not captured by conventional GWAS, providing candidate genes that may contribute to future breeding strategies after further validation.

## Simple Summary

High temperature and humidity can reduce milk production and milk quality in dairy cows, but cows may differ in how strongly they respond because of their genetic background. This study combined milk recording data from Chinese Holstein cows, weather records, and genetic marker data to examine how milk yield, fat percentage, and protein percentage changed under increasing heat stress. Milk yield declined mainly under moderate and severe heat stress, whereas milk fat and protein percentages decreased earlier. We also found genetic markers whose effects changed under different heat stress conditions. These results suggest that genetic evaluation programs could consider heat stress when selecting dairy cows, helping farmers breed animals that maintain production and milk quality in hotter and more humid environments.

## 1. Introduction

Global warming has become one of the most severe environmental challenges facing human society in the 21st century, and its negative impact on agricultural production systems-particularly livestock farming-is becoming increasingly pronounced. Among various livestock and poultry species, dairy cows generate high metabolic heat, are covered in fur, and have limited heat dissipation capacity, making them highly sensitive to high-temperature and high-humidity environments and one of the livestock species most severely affected by heat stress [1]. As the dominant breed in China’s dairy cattle industry, Chinese Holsteins exhibit high milk production but are particularly prone to heat stress, which has become a key bottleneck constraining the sustainable development of China’s dairy industry.

A key mechanism by which heat stress impairs milk production is a decrease in dry matter intake (DMI), which leads to a negative energy balance and a series of metabolic adaptive responses—including changes in rumen fermentation, reduced visceral blood flow, and a shift in nutrient allocation toward thermoregulation rather than milk synthesis [2]. These changes directly affect the supply of precursors for milk fat and protein synthesis, thereby influencing milk yield and composition. Furthermore, genetic differences among individuals in feed efficiency and metabolic flexibility result in varying degrees of DMI reduction and metabolic responses, providing a physiological basis for genotype-environment (G×E) interactions under heat stress conditions.

The Temperature-Humidity Index (THI) is currently the most widely used international metric for quantifying heat stress. By integrating ambient temperature and relative humidity, this index comprehensively reflects the combined impact of environmental factors on dairy cows’ heat dissipation capabilities, addressing the limitation of single-temperature metrics that fail to account for how high humidity exacerbates heat stress. Based on THI values, heat stress is typically classified into four levels: no-heat-stress (THI < 68), mild heat stress (68 ≤ THI < 72), moderate heat stress (72 ≤ THI < 80), and severe heat stress (THI ≥ 80) [3].

Previous studies across different regions and production systems have confirmed the close relationship between THI and dairy cow milk production performance [4]. In a study of Holstein cows in the United States, measured daily milk yield began to decline when the THI reached approximately 72, with milk yield decreasing by about 0.2 kg for every 1-unit increase in THI. Subsequent parameter estimation studies further indicated that the response to heat stress has a heritable basis. Under Mediterranean climatic conditions, when the THI increased from 68 to 78, dairy cows’ milk yield decreased by approximately 21%, and dry matter intake decreased by approximately 9.6%; when the THI exceeded 69, milk yield decreased by approximately 0.41 kg for every 1-unit increase in the THI, while milk fat and protein percentage were lower under high THI conditions in summer compared to lower THI conditions in spring. In recent years, studies based on large-scale records have further demonstrated that different THI indices vary in their ability to explain milk yield and milk composition traits; milk fat and protein percentage may be affected even at lower THI levels [5], whereas milk yield typically shows a significant decline only under higher THI conditions. These studies indicate that the effects of heat stress on milk production traits are reflected not only in reduced milk yield but also in changes in milk composition and quality.

However, dairy cows do not respond uniformly to changes in the thermal environment. Even under identical or similar THI conditions, significant differences exist among individuals in terms of the extent of milk yield decline, milk composition stability, and heat tolerance. In addition to being influenced by feeding management, lactation stage, and physiological status, these differences may also be related to individual genetic background. Previous studies have shown that heat stress responses have a genetic basis, and that heat stress-related genetic parameters and heritabilities can vary with THI levels and parity [6]. These differential responses between genetic backgrounds and thermal environments manifest as gene-environment interactions (G×E) [7]. Traditional GWAS studies primarily focus on the main effects of SNPs on average trait levels, making it difficult to characterize the dynamic patterns of SNP effects as they vary along a continuous THI gradient. For longitudinal data such as daily milk yield records, random regression models can utilize repeated observations under different THI conditions to express genetic effects as functions of environmental variables, making them more suitable for detecting the genetic sensitivity of milk yield traits to changes in the thermal environment [8].

Currently, there are still relatively few studies on Chinese Holstein dairy cows that systematically examine SNP × THI interaction effects at the genome-wide level using longitudinal daily records. In particular, it remains to be further elucidated whether QTLs for classic milk production traits maintain stable effects under different THI conditions [9] and which candidate genes may influence the sensitivity of milk production traits to heat stress [10]. Therefore, identifying environment-dependent genetic loci under a continuous THI gradient not only helps supplement the understanding of the genetic basis of milk production traits obtained from conventional main-effect GWAS but also provides candidate markers for molecular breeding for heat tolerance. This study focuses on Chinese Holstein dairy cows and examines the genetic response patterns of milk production traits under heat stress conditions, aiming to systematically elucidate the mechanisms by which dynamic thermal environments influence dairy production traits from the perspective of gene-environment interactions. Specific research objectives include: (1) quantifying the patterns of change in milk yield, milk fat percentage, and milk protein percentage under different THI levels; (2) using a random regression GWAS model to detect SNP loci with interactive effects with THI across the entire genome; (3) screening and functionally annotating candidate genes associated with heat stress response to provide a theoretical basis for molecular breeding for heat tolerance in dairy cattle. (4) performing separate temperature- and humidity-based GWAS to dissect their relative contributions to the THI-driven signals and to identify loci potentially responding specifically to each stressor.

Compared with previous heat-stress GWAS that typically used discrete two-group comparisons or reaction norm models with fixed THI thresholds [6,7,9], our study offers several improvements. We treated THI as a continuous covariate using Legendre polynomials to capture the full trajectory of SNP effects across the entire THI gradient without subjective thresholding. We also performed parallel temperature- and humidity-based GWAS to dissect their relative contributions, revealing humidity-specific signals that would be masked in a single-index model. Additionally, the near-zero overlap between THI-GWAS and conventional GWAS signals for milk yield suggests that the genetic architecture of heat stress response may differ fundamentally from that of average production performance. These features distinguish our study from previous investigations and provide new insights into the genetic basis of heat tolerance in Chinese Holstein cattle.

## 2. Materials and Methods

### 2.1. Experimental Materials

#### 2.1.1. Phenotypic Data

The initial phenotypic database comprised 20,226,440 records from 406,191 Chinese Holstein dairy cows, collected from July 2004 to December 2021. The records included milk yield (MY), milk fat percentage (FP), and milk protein percentage (PP). All dairy cows on the farm are kept under similar husbandry conditions, including comparable housing, feeding, and milking systems. To ensure the accuracy and reliability of the phenotypic data, we applied the following screening criteria: The screening criteria were as follows: each cow had at least 6 records; only production records from the first two lactations were retained, and cows with data from the second lactation must also have records from the first lactation; the number of lactation days was restricted to between 5 and 305 days; the first testing day must occur within 45 days postpartum, and the interval between two consecutive test days must not exceed 70 days; the age at first calving was restricted to 22–38 months, and for the second calving to 34–50 months; milk yield was restricted to 5–80 kg, milk fat percentage to 1.4–6.2%, and milk protein percentage to 2.0–5.0%. After applying these criteria, 398,951 cows and 20,163,106 records were excluded, leaving a final dataset of 63,334 records from 7240 cows for subsequent analysis.

#### 2.1.2. Genotype Data

**Figure 1:**
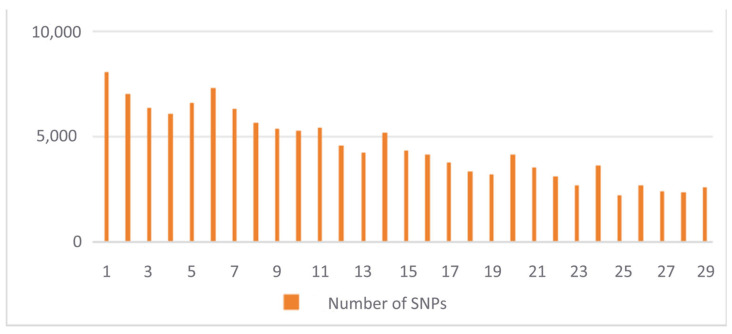
Distribution of quality-controlled SNPs across autosomes.

The genotype data in this study were derived from bovine SNP chips of varying densities, including Illumina Bovine SNP50v1 (50Kv1) (Illumina, Inc., San Diego, CA, USA), Illumina Bovine SNP50v2 (50Kv2) (Illumina, Inc., San Diego, CA, USA), GGP Bovine HD (80K) (Neogen GeneSeek Operations, Lincoln, NE, USA), and GGP Bovine HDv3 (150K) (Neogen GeneSeek Operations, Lincoln, NE, USA). To standardize locus coverage across different chip platforms, this study used the 150K chip locus set as the target and performed genotype imputation and integration of low-density chip data using Beagle v4.1 [11]. This resulted in unified high-density chip genotype data for subsequent GWAS analysis. SNP coordinates were based on the ARS-UCD1.2 reference genome. Quality control of the genotype data was performed using PLINK 1.9 software [12,13], with the following criteria: only SNP loci with clear localization on autosomes were retained; minor allele frequency (MAF) > 0.01; individual genotype missing rate < 0.05; site missing rate < 0.05; and Hardy–Weinberg Equilibrium (HWE) test *p*-value > 1 × 10^−6^. A total of 113,297 SNP loci were ultimately retained for GWAS analysis. The distribution of SNP loci across chromosomes is shown in Figure 1.

#### 2.1.3. Meteorological Data

The meteorological data were obtained from the Institute of Geographic Sciences and Natural Resources Research, Chinese Academy of Sciences. The data cover meteorological observations from 2001 to 2023 in the districts and counties where 72 dairy farms in Beijing, Shanghai, and Shandong are located, including daily average temperature, daily maximum temperature, daily minimum temperature, and daily average relative humidity. The nearest-neighbor matching method was used to link weather station data to each measurement day at each dairy farm (for farms located more than 10 km away, inverse-distance-weighted average interpolation using data from the three nearest stations was applied). The Temperature-Humidity Index (THI) was calculated using the daily average temperature and daily average relative humidity for each measurement day, without accounting for lag effects. In the sensitivity analysis, the THI was replaced with either the daily average temperature or the daily average relative humidity to assess the sensitivity of each factor individually. Following quality control, the complete matching rate between meteorological data on measurement days and milk production performance records reached 99.7%. Figure S1 shows the trends in daily maximum and minimum temperatures in the three regions of Beijing, Shanghai, and Shandong from 2001 to 2023; the solid line represents the daily maximum temperature, and the dashed line represents the daily minimum temperature.

### 2.2. Data Analysis

#### 2.2.1. THI Calculation Formula

To comprehensively characterize the thermal load level of the dairy cows’ environment, this study adopted THI as the primary environmental indicator for evaluating heat stress intensity. The specific calculation formula is as follows:

$$THI=\left(1.8\times T_{air}+32\right)-\left(0.55-0.55\times RH\right)\times \left(1.8\times T_{air}-26\right)$$

where Tair is the ambient temperature (°C) and RH is the relative humidity (expressed as a decimal, with a range of 0–1). Based on the calculated THI values, the heat stress status for each measurement day was classified as no-heat-stress (THI < 68), mild heat stress (68 ≤ THI < 72), moderate heat stress (72 ≤ THI < 80), and severe heat stress (THI ≥ 80) [3].

#### 2.2.2. Random Regression Model

To investigate the dynamic patterns of SNP effects as THI changes, this study employed the random regression model proposed by Ning et al. for longitudinal GWAS analysis [14], which is implemented in the GMAT 2 software (https://github.com/chaoning/GMAT, accessed on 15 March 2025). The specific model is as follows:

$$y_{ijn}(t)=htd_{i}+\overset{n_{f1}}{\underset{k=0}{\sum }}\beta_{jk}\varphi_{k}(t)+\overset{n_{f2}}{\underset{k=0}{\sum }}\alpha_{k}\chi_{nm}\varphi_{k}(t)+\overset{n_{a}}{\underset{k=0}{\sum }}a_{nk}\varphi_{k}(t)+\overset{n_{p}}{\underset{k=0}{\sum }}P_{nk}\varphi_{k}(t)+e_{nt}$$

where $y_{ijn}\left(t\right)$ is the recorded value (milk yield, milk fat percentage, or milk protein percentage) for individual n at environmental variable t (THI, temperature, or humidity); $htd_{i}$ is the fixed effect of the i-th herd-test-day; $\beta_{jk}$ is the k-th regression coefficient for the population mean of the j-th calving year-season; $\alpha_{k}$ is the k-th regression coefficient for the effect of the m-th SNP; $\chi_{nm}$ is the genotype of the n-th individual at the m-th SNP (coded as 0, 1, or 2); $a_{nk}$ is the k-th regression coefficient for the additive genetic effect of individual n; ${Ρ}_{nk}$ is the k-th regression coefficient for the permanent environmental effect of individual n; $e_{nt}$ is the random residual; and $\varphi_{k}\left(t\right)$ is the k-th order normalized Legendre polynomial basis function evaluated at t. In this study, a fourth-order Legendre polynomial (k = 0, 1, 2, 3, 4) was used for all random regression terms. To improve numerical stability, the environmental variable t was standardized to the interval [−1, 1] prior to analysis using the transformation $\chi =2\left(t-t_{\min}\right)/\left(t_{\max}-t_{\min}\right)-1$.

For the random effects, the additive genetic effect vector $a=\left[a_{n}\left(t\right)\right]$ follows a multivariate normal distribution a~Ν (0, G⊗${Κ}_{a}$), where G is the genomic relationship matrix constructed from SNP markers and ${Κ}_{a}$ is the additive genetic covariance matrix. The permanent environmental effect vector $p=\left[p_{n}\left(t\right)\right]$ follows p~N (0, I⊗${Κ}_{P}$), where ${Κ}_{P}$ is the permanent environmental covariance matrix. The random residuals $e_{nt}$ are assumed to be independently and identically distributed as e~N (0, $Ι\sigma_{e}^{2}$). Variance components were estimated using the weighted expectation maximization and average information algorithm [14], implemented in the GMAT software.

Standardization of Environmental Variables and Legendre Polynomial Fitting: To eliminate the influence of the dimensionality of environmental variables and improve the numerical stability of the polynomial fitting, the raw environmental variable t (with a range of [t_min, t_max]) is first linearly transformed to the standardized interval [−1, 1] as follows:

$$\chi =2\left(t-t_{\min}\right)/\left(t_{\max}-t_{\min}\right)-1$$

Based on this, normalized Legendre polynomial basis functions are used to fit $\mu \_j\left(t\right)$, $SNP\_m\left(t\right)$, $a\_n\left(t\right)$, and $p\_n\left(t\right)$:

$$\varphi_{n}(\chi )=\sqrt{\frac{2n+1}{2}}•P_{n}(\chi )$$

where ${Ρ}_{n}\left(\chi \right)$ is the nth-order Legendre polynomial, with *n* = 0, 1, 2, 3, 4 (this study uses fourth-order fitting). Normalization ensures that the basis functions are orthogonal on the interval [−1, 1].

Variance Component Estimation: To speed up computations, variance components are first estimated in a baseline model that does not include fixed effects for individual SNPs, and these are then applied to the analysis of all subsequent SNP loci. Variance component estimation uses the weighted expected maximization and average information algorithm [14].

Significance Test for SNP × THI Interactions: For each SNP, a Wald test is used to assess whether its Legendre coefficient vector is significantly different from zero. Specifically, the SNP effect can be expressed as $\beta_{0}\varphi_{0}\left(t\right)+\beta_{1}\varphi_{1}\left(t\right)+\beta_{2}\varphi_{2}\left(t\right)+\beta_{3}\varphi_{3}\left(t\right)+\beta_{4}\varphi_{4}\left(t\right)$, where $\beta_{0}$ represents the mean main effect, and $\beta_{1}-\beta_{4}$ represent THI-dependent dynamic effects; the null hypothesis for the SNP × THI interaction test in this study is $\beta_{1}$ = $\beta_{2}$ = $\beta_{3}$ = $\beta_{4}$ = 0, and the Wald statistic approximately follows a $\chi^{2}$ distribution with 4 degrees of freedom. This method simultaneously tests the overall significance of SNP effects as they vary with environmental variables, rather than testing only at a single time point, thereby effectively controlling the risk of inflation from multiple comparisons.

The Bonferroni method was used for multiple comparison correction. However, applying the standard Bonferroni correction directly to all 113,297 SNPs would be overly conservative because it assumes independence among all tested markers, ignoring the linkage disequilibrium (LD) structure of the genome, where adjacent SNPs are often correlated. To account for LD among SNP loci while maintaining statistical power, we performed LD-based pruning using PLINK software with the following parameters: a sliding window of 100 kb, a step size of 50 SNPs, and an r^2^ threshold of 0.2 [15]. This approach identifies a set of approximately independent SNP markers by removing SNPs that are in strong LD (r^2^ > 0.2) with neighboring markers within each window. After LD pruning, approximately 12,280 independent SNP loci were retained. The effective number of independent tests was thus estimated as 12,280, and the genome-wide significance threshold was calculated as α = 0.05/12,280 ≈ 4.07 × 10^−6^. This threshold balances the control of family-wise error rate at α = 0.05 with the preservation of detection power for true associations, and is consistent with widely used practices in livestock GWAS [15,16,17].

Comparative analysis models: To evaluate the robustness of the THI-based model and explore the individual roles of temperature and humidity, this study established three types of analyses: (1) A temperature-interaction GWAS model with the same structure as the THI-GWAS, except that ‘t’ was replaced with daily average temperature; (2) a humidity-interaction GWAS model, with ‘t’ replaced by daily average relative humidity. (3) Conventional main-effect GWAS, with the model form $y\_\left[ijn\right]=htd\_i+\mu \_j+\chi \_\left[nm\right]\cdot SNP\_m+a\_n+p\_n+e\_\left[nt\right]$, where all effects are constants rather than functions of environmental variables, and the model does not include any environmental variables or their interaction terms with SNPs.

#### 2.2.3. Descriptive Comparison of Milk Production Traits Under Different Heat Stress Levels

To describe the trends in milk production traits under different heat stress levels, the records corresponding to each THI level were classified into four groups based on heat stress level: no-heat-stress, mild heat stress, moderate heat stress, and severe heat stress. The weighted means, pooled standard deviations, and standard errors for milk yield, milk fat percentage, and milk protein percentage were calculated within each group. To assist in comparing phenotypic differences among the different heat stress levels, Welch’s *t*-test was further used for pairwise comparisons. Welch’s *t*-test does not require homogeneity of variances among groups and is suitable for situations where variances between groups may differ. A significance threshold of 0.05/6 = 0.0083 was used for pairwise comparisons. All *p*-values reported below are Bonferroni-corrected *p*-values. Significance levels are denoted as follows: *** *p* < 0.001, ** *p* < 0.01, * *p* < 0.05.

#### 2.2.4. Candidate Gene Annotation

To elucidate the potential biological functions of significant SNP loci, this study performed candidate gene annotation on the genome-wide significant SNPs identified by the THI-GWAS. The annotation workflow is as follows: Genome version: The bovine reference genome ARS-UCD1.2 and annotation information from Ensembl release 109 were used. Sources of annotation information: Gene location and functional annotations were based on the Ensembl genome database (release 109, downloaded in April 2023), with cross-validation performed using NCBI’s RefSeq database. Definition of annotation window: A genomic interval extending 500 kb upstream and downstream from the physical location of each significant SNP (lead SNP). For merged QTL regions, the entire QTL interval (with a 500-kb extension on both sides after merging) was used as the annotation window.

Candidate gene screening criteria: Any gene falling within the aforementioned annotation window is considered a candidate gene; if the window contains multiple genes, all are included in the candidate gene list; no single “nearest gene” selection is performed to preserve more comprehensive biological information.

Identification of multi-trait shared genes: A gene is identified as a multi-trait shared candidate gene if it appears within the annotation window of a significant SNP in the THI-GWAS analyses for two or three milk production traits.

To standardize terminology, this study distinguishes between the following concepts: “Significant SNPs” refer to all SNP loci that meet the genome-wide significance threshold (*p* < 4.07 × 10^−6^); “lead SNP” refers to the significant SNP with the smallest *p*-value within the same QTL region, used to represent the strongest association signal in that region. For QTL regions containing only a single significant SNP, that SNP is designated as the lead SNP.

#### 2.2.5. Definition of Candidate Signals with Strong Statistical Support

To identify environment-dependent genetic loci that are more reliably influenced by thermal environments, this study conducted a tiered evaluation of candidate genes based on statistical evidence from the THI-interaction GWAS and the two sensitivity analyses (temperature-interaction GWAS and humidity-interaction GWAS):

(1) Core candidate genes: SNPs that reached genome-wide significance (*p* < 4.07 × 10^−6^) in the THI-GWAS and achieved nominal significance (*p* < 0.05) in both the temperature interaction GWAS and the humidity interaction GWAS. This criterion requires the SNP effect to be statistically supported across all three environmental dimensions—THI, temperature, and humidity—and aims to identify genetic loci that are most robustly influenced by the thermal environment.
(2) Potential candidate genes: SNPs that reached genome-wide significance in the THI-GWAS and achieved nominal significance (*p* < 0.05) in at least one of the two sensitivity analyses (temperature or humidity GWAS), but did not meet the criteria for Core candidate genes (i.e., did not reach nominal significance in both sensitivity analyses simultaneously). These genes show moderate evidence of environment-dependent effects and warrant further investigation.
(3) Suggestive candidate genes: SNPs that reached genome-wide significance in the THI-GWAS but did not achieve nominal significance (*p* < 0.05) in either the temperature interaction GWAS or the humidity interaction GWAS. Evidence of environmental dependence for these genes is relatively insufficient and requires further validation through independent populations or functional experiments.

It should be emphasized that this classification is based solely on statistical evidence from the current dataset and does not represent a definitive ranking of biological or functional importance. The actual roles of all candidate genes—regardless of tier—require validation through subsequent molecular biology experiments and independent replication studies.

## 3. Results

### 3.1. Data Overview and Phenotypic Distribution

**Figure 2:**
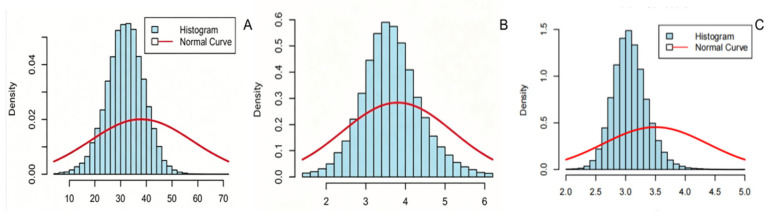
The phenotypic distribution of the number of records for milk production traits. Note: (**A**) MY; (**B**) FP; (**C**) PP.

Following rigorous screening and quality control, a total of 7240 Holstein dairy cows were included in the subsequent analysis, comprising 63,334 records. Descriptive statistics for each milk production trait are presented in Table 1. The mean milk yield per test day was approximately 32.11 kg, with a range of 5.00–71.50 kg; the average milk fat percentage was approximately 3.68%, with a range of 1.40% to 6.20%, and the average milk protein percentage was approximately 3.11%, with a range of 2.00% to 5.00%. Histograms show that the records for all three milk production traits exhibited approximately unimodal distributions, with relatively concentrated overall distributions (Figure 2), and no significant skewness was observed in the data distributions.

The distribution of records across different THI ranges is as follows: the no-heat-stress group had a total of 43,344 records (68.4%), the mild heat-stress group had 5876 records (9.3%), the moderate heat-stress group had 12,708 records (20.1%), and the severe heat-stress group had 1406 records (2.2%). Each category contained a sufficient number of records.

### 3.2. Relationship Between THI and Phenotypic Responses of Milk Production Traits

**Figure 3:**
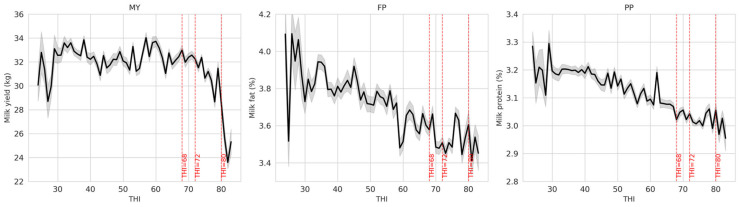
Descriptive Trajectories of Milk Production Traits across the THI Gradient. Note: Shaded areas show 95% CIs for fitted means.

**Figure 4:**
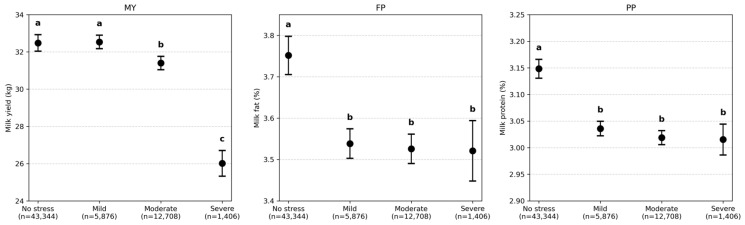
Mean Milk Performance across Heat-Stress Categories. Note: Error bars correspond to 95% confidence intervals. Different lowercase letters above the error bars indicate significant differences between categories (*p* < 0.05, Welch’s *t*-test with Bonferroni correction).

To evaluate the impact of thermal environmental changes on the milk production performance of Chinese Holstein cows, this study analyzed the trends in milk yield, milk fat percentage, and milk protein percentage under different THI levels (Figure 3). The results showed that as THI increased, all three milk production traits exhibited an overall downward trend. Milk yield fluctuated minimally when THI was below approximately 74, but subsequently showed a more pronounced downward trend as THI continued to rise; milk fat and protein percentages exhibited a continuous, gradual decline with increasing THI. Further tests for significant differences by heat stress level were conducted (Figure 4) using Welch’s *t*-test with Bonferroni correction for multiple comparisons (setting the significance threshold for pairwise comparisons at 0.05/6 = 0.0083). As shown in Figure 4, the 95% confidence intervals for MY indicated clear separation between the no-stress/mild groups (≈33.1 kg) and the moderate (≈31.6) and severe (≈26.0 kg) groups, confirming that MY declined substantially only under moderate and severe heat stress. For FP, the no-stress group showed a slightly higher mean (3.78%) than the three stress categories (3.52–3.72%), but the differences among the stress groups were small. For PP, a gradual downward trend was observed from no-stress (3.15%) to severe stress (2.95%), with the largest drop occurring between no-stress and mild stress. Detailed pairwise statistical results are provided in Tables S1–S3.

### 3.3. THI Interaction GWAS Based on a Random Regression Model

#### 3.3.1. Overall Results of the THI-GWAS and Candidate QTL Regions

To analyze the genetic response of milk production traits to changes in the thermal environment, THI was used as a continuous environmental variable, and a random regression GWAS model was employed to detect SNP × THI interaction effects. A total of 149 significant SNP × THI interactions were detected, including 5, 86, and 58 for milk yield, milk fat percentage, and milk protein percentage, respectively. Compared with conventional main-effect GWAS, THI-GWAS identified more SNP-trait associations for milk fat percentage and milk protein percentage (THI-GWAS vs. conventional main-effect: 86 vs. 44 for milk fat percentage; 58 vs. 49 for milk protein percentage). Centered on the physical location of each significant SNP, genomic regions extending 500 kb upstream and downstream were defined, and all annotated genes within these regions were identified as candidate genes for that SNP locus. From a genome-wide distribution perspective, significant signals were primarily concentrated in regions such as BTA5, BTA6, BTA14 and BTA20. Among these, BTA14 exhibited a signal region, where significant or strong THI interaction signals were detected across multiple milk production traits. QTL Region Delineation: SNP loci meeting the genome-wide significance threshold of *p* < 4.07 × 10^−6^ were selected. Subsequently, the significant SNPs were sorted by chromosome number and physical location. On the same chromosome, if the physical distance between two adjacent significant SNPs was less than 1 Mb, they were merged into a single QTL region. Based on the delineation of the above regions, each region was expanded by 500 kb on either side to define the QTL regions. As a result, a total of 13 QTL regions were identified that reached the genome-wide significance level (*p* < 4.07 × 10^−6^). Detailed information on each region is provided in Table 2; the table lists only representative results for the major QTL regions and does not include all significant SNPs. All listed SNPs reached the genome-wide significance level. Note: The minimum *p*-values listed in the table are all results from the SNP × THI interaction test (H_0_: β_1_ = β_2_ = β_3_ = β_4_ = 0) in the THI-GWAS. For comparison, the *p*-value for the main effect of the representative locus in the *DGAT1* region (*ARS-BFGL-NGS-4939*) on milk fat percentage in the conventional main-effect GWAS was 1.19 × 10^−68^.

BTA14 region: Significant SNP loci on this chromosome can be divided into three QTL regions. Region 1 (1–4,959,231 bp, approximately 4.96 Mb): This region was constructed by merging adjacent significant SNPs separated by a physical distance of less than 1 Mb (with a 500-kb extension on either side after merging). A total of 75, 11, and 5 significant SNPs were detected for milk fat percentage, milk protein percentage, and milk yield, respectively, covering the *DGAT1*, *CPSF1*, and *LOC787350* genes. Table 2 lists the representative SNPs and candidate gene information for each trait in this region. Region 2: Covers the *LOC112449570* gene; a significant signal was detected only for milk fat percentage. Region 3: Covers the *KCNS2* gene; a significant signal was detected only for milk protein percentage. Note: According to the merging rule (based on the physical distance between adjacent significant SNPs), a total of 3 independent QTL regions were identified on BTA14. Within each QTL region, the interval was subsequently expanded by 500 kb on both sides to define the candidate gene annotation window. Notably, after this 500-kb expansion, the nearest boundaries of Region 1 and Region 2 are separated by approximately 0.20 Mb (197,757 bp). However, because the underlying significant SNPs defining each region are separated by more than 1 Mb, they are retained as independent QTL regions.

BTA5 Region: A total of 10 significant SNP loci were detected on this chromosome, which can be divided into two QTL regions. Region 1 (4,017,412–5,017,412 bp) covers the *KCNC2* gene, with only one significant SNP detected for milk protein percentage; Region 2 (92,561,021–94,669,217 bp) covers the *MGST1*, *EPS8*, and *SLC15A5* genes; 9 significant SNPs were detected for milk fat percentage, with relatively strong signals.

BTA20 Region: A total of 20 significant SNP loci were detected on this chromosome, which can be divided into three QTL regions, all of which are associated with milk protein percentage. Among these, BTA20_region1 (30,728,123–34,309,571 bp) covers the *FRMD4B* and *FAM19A1* genes and contains 18 significant SNPs; BTA20_region2 (36,400,514–37,400,514 bp) covers the *WDR70* gene and contains 1 significant SNP; BTA20_region3 (39,396,176–40,396,176 bp) covers the *ADAMTS12* gene and contains 1 significant SNP.

**Figure 5:**
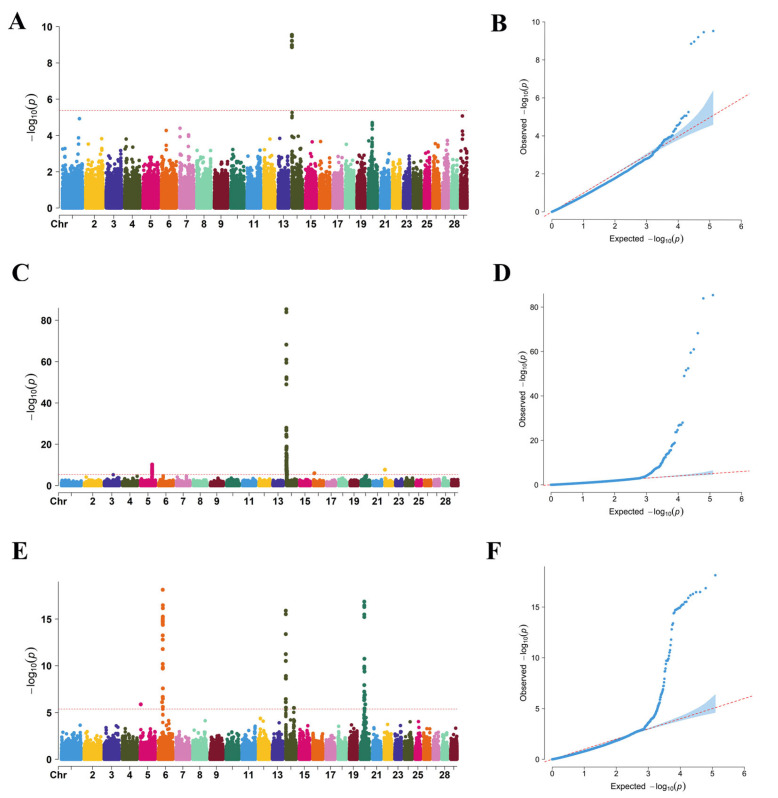
Manhattan and QQ plots of SNP × THI interaction GWAS for milk production traits. Note: (**A**,**B**) MY; (**C**,**D**) FP; (**E**,**F**) PP. The red horizontal line indicates the Bonferroni correction threshold.

Other chromosomal regions: Two QTL regions were detected on BTA6 (covering *GRID2* and *PKD2*, both associated with milk protein percentage), and one region was detected on BTA16 (*LOC101903754*, milk fat percentage). Manhattan plots show that the significant loci in the above QTL regions all reached the genome-wide significance level after Bonferroni correction (*p* < 4.07 × 10^−6^), and QQ plots show a marked deviation at the tails for each trait, suggesting the presence of several strongly associated loci (Figure 5). See Tables S4 and S5 for details.

#### 3.3.2. Sharing and Specificity of THI Interaction Signals Across Different Milk Production Traits

A comparison of significant signals across three milk production traits showed that most associations were trait-specific, and a limited number of regions were shared between two or more traits. The highest number of significant SNPs was detected for milk fat percentage. Signals for milk protein percentage were distributed across BTA14, BTA6, and BTA20. The number of significant SNPs detected for milk yield was relatively low and primarily located on BTA14.

Shared QTL regions: BTA14 was shared as a candidate region among the three traits, containing candidate genes closely associated with milk production traits, such as *DGAT1* and *CPSF1*, and exhibiting THI interaction signals to varying degrees in milk fat percentage, milk protein percentage, and milk yield. Among these, the *DGAT1* region showed the strongest signal for milk fat percentage and also reached significant levels for milk yield and milk protein percentage; the *CPSF1* region showed significant signals for milk yield and milk protein percentage.

Specific QTL regions: QTL regions specific to milk fat percentage included the *MGST1* region on BTA5, the *LOC112449570* region on BTA14, the *LOC101903754* region on BTA16, and the *LOC787350* region on BTA14. Significant QTL regions specific to milk protein percentage included the *KCNC2* region on BTA5; the *GRID2* and *PKD2* regions on BTA6 (24 SNPs); the combined *FRMD4B*/*FAM19A1* region on BTA20; and the *WDR70* and *ADAMTS12* regions. No specific QTL regions were detected for milk yield. In contrast, the conventional main-effect GWAS identified only one significant SNP for milk yield, far fewer than the THI-GWAS (5 SNPs), while milk fat percentage (86 significant SNPs in the THI-GWAS versus 44 in the conventional GWAS) and milk protein percentage (58 significant SNPs in the THI-GWAS versus 49 in the conventional GWAS) also showed that the THI-GWAS detected more significant SNPs. See Tables S4 and S5 for details.

#### 3.3.3. THI-Dependent Effects of Key Candidate Regions and Candidate Genes

**Figure 6:**
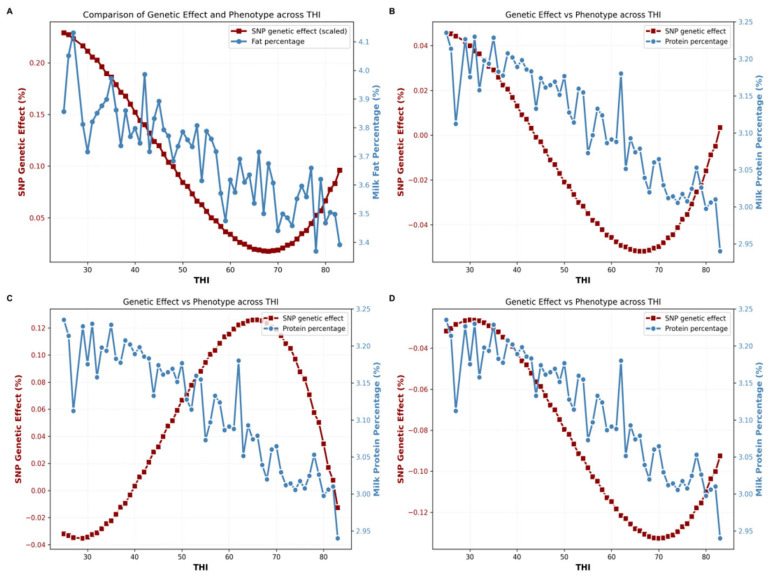
Dynamic genetic effects of representative significant SNPs across the THI gradient. Note: (**A**) SNP ARS-BFGL-NGS-4939 (Chr14); (**B**) BovineHD1400018987 (Chr14); (**C**) ARS-BFGL-NGS-26909 (Chr20); (**D**) SNP UFL-rs134432442 (Chr14).

Among the significant signals detected in the THI-GWAS, the BTA14 region stood out the most. This region contains classic candidate genes in dairy cow milk production trait research, such as *DGAT1* and *CPSF1*. Loci near *DGAT1* exhibited a very strong SNP × THI interaction effect on milk fat percentage. The effect curve of a representative SNP (Figure 6) shows that its genetic effect on milk fat percentage is stronger under lower THI conditions and gradually weakens as THI increases. Similarly, the effect of representative SNPs in the *CPSF1* region on milk yield also varies along the THI gradient (shifting from negative to positive and crossing the zero point at a THI of approximately 75).

Based on the regions containing significant SNPs and the annotation of neighboring genes, this study identified a total of 52 candidate genes associated with THI interaction effects. In addition to the aforementioned *DGAT1*, *CPSF1*, and *MGST1*, these include genes such as *ABCG2*, *VPS28*, and *PPP1R16A*. Among these, *DGAT1* and *CPSF1* showed associations with multiple milk production traits, while the others were trait-specific. Furthermore, this study identified six candidate genes with limited prior evidence (*ZNF250*, *GRID2*, *LOC112449570*, *LOC112449590*, *LOC101903754*, *LOC787350*). By contrast, *EPS8*, also located in BTA5_region2, was not included in this list because it has been previously reported in the literature as being associated with milk production traits in dairy cattle [18,19].

### 3.4. Analysis of Temperature and Humidity Sensitivity

#### 3.4.1. Correlation Among THI, Temperature, and Humidity

To assess multicollinearity among the Heat Stress Index (THI), temperature (T), and relative humidity (RH), we used each meteorological record (63,334 in total) as the basic unit of analysis and calculated the Pearson correlation coefficients among these three variables. The results showed that temperature and THI exhibited a very strong positive correlation (r = 0.99, *p* < 0.001), indicating that temperature is the key determinant of THI levels; relative humidity and THI exhibited a significant, moderate positive correlation (r = 0.41, *p* < 0.001); there was also a certain degree of positive correlation between temperature and relative humidity (r = 0.14, *p* < 0.001), indicating that, within the scope of the data in this study, hot weather is associated with higher relative humidity.

#### 3.4.2. Comparison of Temperature Interaction GWAS Results with THI Results

**Figure 7:**
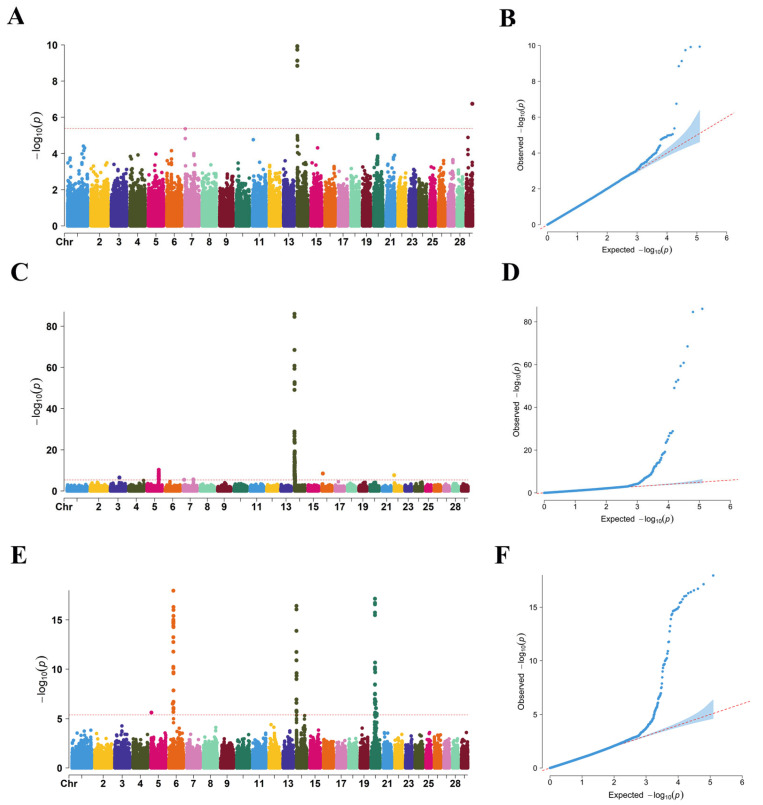
Manhattan and QQ plots of SNP × temperature interaction GWAS for milk production traits. Note: (**A**,**B**) MY; (**C**,**D**) FP; (**E**,**F**) PP. The red horizontal line indicates the Bonferroni correction threshold.

The results of the temperature-interaction GWAS are shown in Figure 7. A total of 161 significant SNP loci associated with temperature variation were identified, with 6, 93, and 62 detected in milk yield, milk fat percentage, and milk protein percentage, respectively. The temperature GWAS and THI-GWAS showed high consistency in the major significant chromosomes, particularly in the BTA14 region, as well as in some candidate regions. Strong association signals were also detected in the BTA14 region in the temperature GWAS (minimum *p* = 8.89 × 10^−87^), and the trends in the effect curves of representative loci were similar to those observed in the THI analysis. See Table S7 for details.

#### 3.4.3. Comparison of Humidity Interaction GWAS Results with THI Results

**Figure 8:**
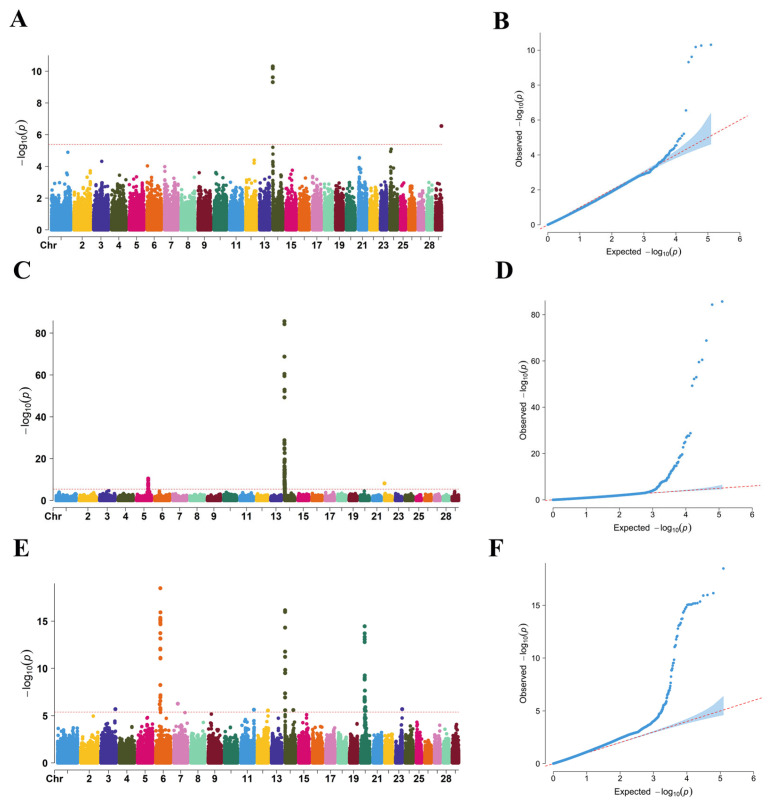
Manhattan and QQ plots of SNP × relative humidity interaction GWAS for milk production traits. Note: (**A**,**B**) MY; (**C**,**D**) FP; (**E**,**F**) PP. The red horizontal line indicates the Bonferroni correction threshold.

The results of the humidity-based GWAS are shown in Figure 8. A total of 159 significant SNP loci were associated with changes in humidity, with 6, 91, and 62 detected in milk yield, milk fat percentage, and milk protein percentage, respectively. The humidity-interaction GWAS partially overlapped with the THI-GWAS, mainly in BTA14 and BTA20. However, the humidity GWAS also revealed several specific signals (e.g., some loci on BTA5 and BTA16 were significant only in the humidity GWAS). See Table S8 for details.

### 3.5. Conventional Main-Effect GWAS Excluding THI Dynamic Effects and Interaction Terms

**Figure 9:**
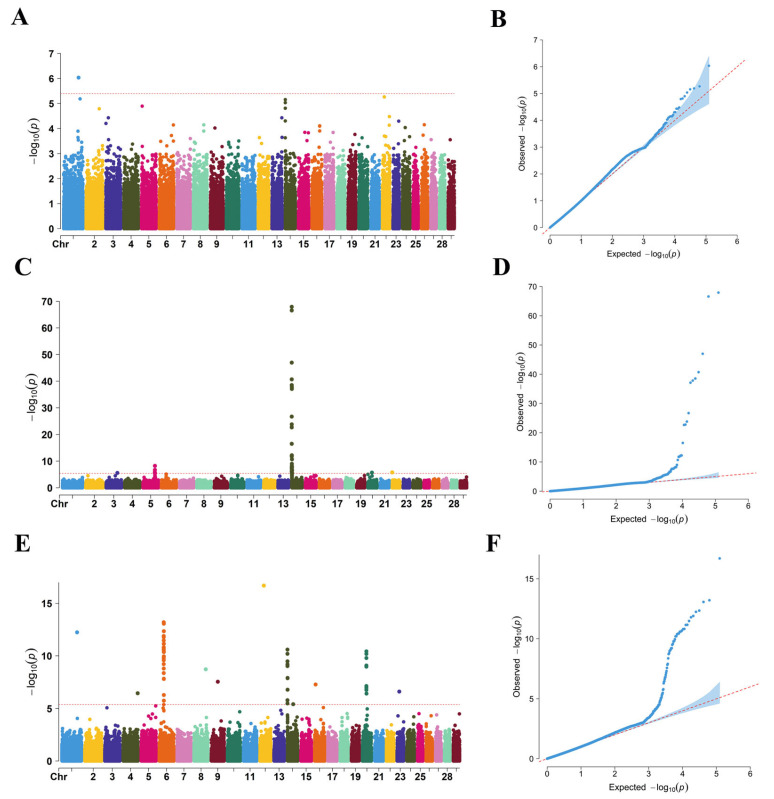
Manhattan and QQ plots of conventional main-effect GWAS for milk production traits. Note: (**A**,**B**) MY; (**C**,**D**) FP; (**E**,**F**) PP. The red horizontal line indicates the Bonferroni correction threshold.

To assess the necessity of including THI dynamic interaction effects, this study conducted a conventional main-effect GWAS as a control analysis; this model did not include THI environmental variables or their interaction terms with SNPs. The results show (Figure 9) that the conventional main-effect GWAS detected a total of 94 significant SNP loci at the genome-wide significance level. Among these, milk yield (MY), milk fat percentage (FP), milk protein percentage (PP) yielded 1, 44, and 49 significant SNPs, respectively. Compared with the THI-GWAS results (a total of 149 significant SNP-trait associations), the conventional main-effect GWAS detected fewer associations for milk yield (1 vs. 5) and notably fewer for milk fat percentage and milk protein percentage. A further comparison of the overlap between the two analyses revealed:

Milk yield: Of the 1 significant SNP detected by the conventional main-effect GWAS, 0 (0%) overlapped with the significant SNPs from the THI-GWAS;

Milk fat percentage: Of the 44 significant SNPs identified by the conventional main-effect GWAS, 40 (90.9%) overlapped with the significant SNPs from the THI-GWAS, primarily concentrated in the *DGAT1* region on BTA14 and the *MGST1* region on BTA5;

Milk protein percentage: Of the 49 significant SNPs identified by the conventional main-effect GWAS, 41 (83.7%) overlapped with the significant SNPs from the THI-GWAS, distributed across the *PKD2* region on BTA6, the *DGAT1* region on BTA14, and the *FRMD4B* region on BTA20.

In the conventional main-effect GWAS, loci near *DGAT1* (such as *ARS-BFGL-NGS-4939*) exhibited highly significant association signals (minimum *p*-value of 1.19 × 10^−68^). The effect curve shows that its genetic contribution to milk fat percentage gradually weakens as THI increases (Figure 6A). See Tables S9 and S10 for details.

### 3.6. Summary of Candidate Genes

Based on the ARS-UCD1.2 reference genome annotation, candidate gene annotation was performed by centering on the genome-wide significant SNPs (*p* < 4.07 × 10^−6^) identified by the THI-GWAS and extending 500 kb upstream and downstream. A total of 52 candidate genes located in the vicinity of the THI-GWAS significant SNPs were identified (Table S6). According to the tiering criteria defined in Section 2.2.5, 50 genes were classified as core candidate genes (including *DGAT1*, *CPSF1*, *ABCG2*, *KCNC2*, and others that were supported by both temperature and humidity sensitivity analyses), one gene (*KCNS2*) was classified as a potential candidate gene (supported by humidity but not temperature analysis), and one gene (*LOC112449570*) was classified as a suggestive candidate gene (not supported by either sensitivity analysis). Regarding published research on heat stress or THI interactions, among the 52 candidate genes, 23 have been previously reported in the literature, 6 have been reported less frequently (*ZNF250*, *GRID2*, *LOC112449570*, *LOC112449590*, *LOC101903754*, and *LOC787350*), and one additional gene (*KCNC2*) was also rarely reported but met the statistical criteria for a core candidate gene, while the remaining 22 are not found in the literature reviewed in this study (Table S6). The literature evidence for these candidate genes is summarized in Table S6 [20,21,22,23,24,25,26,27].

## 4. Discussion

### 4.1. Effects of THI on Milk Production Traits and Their Physiological Basis

This study found that elevated THI had a negative impact on milk yield, milk fat percentage, and milk protein percentage, although the response thresholds and patterns differed among these three traits. The phenotypic patterns (Figure 3 and Figure 4) revealed trait-specific responses to heat stress. Milk yield remained relatively stable under no-stress (≈33.3 kg) and mild (≈32.8 kg) conditions but showed a marked reduction under moderate (≈31.6 kg) and particularly severe stress (≈26.0 kg), suggesting a threshold effect around THI 72–74. In contrast, milk fat percentage decreased only slightly from no-stress (3.72%) to mild/moderate (3.72% and 3.58%)~3.62%. and then plateaued (severe: 3.52%), indicating that fat content is most sensitive during the early phase of heat stress. Milk protein percentage showed a gradual decline across all stress categories (from 3.15% to 2.98%), with the difference between moderate and severe stress being relatively small, implying a cumulative but diminishing effect at higher THI levels.

The physiological basis for the decline in milk yield involves a negative energy balance resulting from reduced feed intake, a decrease in the supply of milk fat precursors due to changes in ruminal fermentation patterns [28], the metabolic reallocation of nutrients toward body temperature maintenance [2], and the direct suppression of gene expression related to milk synthesis in the mammary gland by heat stress. Milk fat percentage decreases significantly in the early stages of heat stress, which is related to the high dependence of milk fat synthesis on feed intake and energy metabolism status. These findings are consistent with previous studies that identified fat digestion and absorption pathways as key biological processes underlying milk fat percentage variation [29], suggesting that heat stress may compromise milk fat synthesis by disrupting these pathways. Milk protein percentage continues to decline from the mild heat stress stage through the moderate heat stress stage, possibly reflecting a progressive inhibition of mammary protein synthesis resulting from suppressed mTOR pathway activity and reduced amino acid utilization efficiency. In addition to the direct physiological effects of heat stress, milk yield and milk composition are also significantly influenced by nutritional management. Although this study did not collect individual-level diet data from each farm, differences among farms in diet formulation, feeding strategies, and dry matter intake may, to some extent, explain part of the phenotypic variation observed under different THI conditions. Future studies that incorporate detailed nutritional records will help distinguish the effects of feeding management from genetic and environmental effects.

### 4.2. THI-GWAS Reveals Environment-Dependent Genetic Effects on Milk Production Traits

The comparison between THI-GWAS and conventional main-effect GWAS should be interpreted with caution, as the two approaches test different hypotheses. Conventional GWAS tests the average SNP effect across environments, whereas THI-GWAS tests whether SNP effects vary along the THI gradient. Thus, the additional associations identified by THI-GWAS reflect environment-dependent signals that are not detectable under the conventional main-effect framework, rather than indicating greater statistical power. Traditional GWAS assumes that SNP effects remain constant across different environmental conditions, which may overlook environment-dependent loci. This study employed a random regression GWAS framework, treating THI as a continuous environmental covariate and using Legendre polynomials to fit the dynamic trajectories of SNP effects as a function of THI. The advantages of this method include: no prior discrete grouping of THI, thereby avoiding subjectivity in threshold selection; the ability to characterize the fine-scale patterns of effects as THI varies continuously; and the ability to calculate SNP effect values and genetic variation patterns at different THI levels.

Based on the THI-GWAS, this study identified a total of 149 SNP-trait associations with interactive effects on THI (reaching genome-wide significance, *p* < 4.07 × 10^−6^), whereas conventional main-effect GWAS detected only 94 significant SNPs, with notably lower numbers for milk fat percentage and milk protein percentage. The difference in the number of significant SNPs detected between the two approaches, particularly the low overlap for milk yield (0%), suggests that the environmentally dependent genetic bases of different traits may differ fundamentally. Milk yield may be influenced by relatively few THI-dependent genetic loci, and its heat stress response may be mediated more by non-genetic factors or a small number of major-effect loci. For milk fat percentage and milk protein percentage, the high overlap rates (90.9% and 83.7%, respectively) indicate that many loci with strong main effects also exhibit environment-dependent effects.

Taking *DGAT1* as an example, conventional GWAS confirmed its strong average main effect (*p* = 1.19 × 10^−68^), while the THI-GWAS further revealed that this effect is not constant but dynamically weakens as THI increases (*p* = 4.29 × 10^−86^ for the dynamic interaction test), and the effect curve showed that its contribution to milk fat percentage gradually weakened as THI increased. Signals were most concentrated in the BTA14 region. Effect curves for top SNPs showed that the effect values of multiple loci exhibited nonlinear changes with increasing THI; for some SNPs, the effect only increased or changed direction after THI rose, suggesting that certain genetic loci may only express their genetic effects under specific environmental stresses. For example, the effect of *DGAT1* on milk fat percentage gradually weakens as THI increases (Figure 6A), while the effect of *CPSF1* on milk yield changes from negative to positive at a THI of approximately 75 (Figure 6D). Several possible explanations may account for the contrast between conventional and THI-GWAS results: (1) The two models differ in their testing objectives and degrees of freedom; (2) The field-testing-day effect may have absorbed some of the differences in environmental and population structure, thereby weakening the detection power of the conventional main-effect model; (3) *DGAT1*’s contribution to milk fat percentage is environment-dependent; its genetic effect is stronger under thermoneutral conditions and gradually weakens as the severity of heat stress increases. These explanations require further research to verify. By enabling the detection of SNP effects that vary continuously with environmental gradients, random regression GWAS provides a powerful tool for elucidating the genetic basis of environmental adaptability in complex traits—complementing rather than replacing conventional main-effect GWAS.

The random regression GWAS employed here offers advantages over conventional main-effect GWAS and reaction norm models with fixed thresholds [6,9], as it fits continuous effect trajectories without prior discretization. This is particularly relevant for milk yield, where we observed no overlap between THI-GWAS and conventional GWAS signals. Previous approaches may overlook loci with nonlinear allelic effects along environmental gradients. We suggest that similar frameworks could benefit future studies on environmental adaptability in other livestock species and stressors.

### 4.3. Major Candidate Genes and Their Potential Mechanisms

Based on the regions containing significant SNPs and the annotation of neighboring genes, this study identified a total of 52 candidate genes associated with THI interaction effects. These genes can be categorized into two groups based on the strength of prior evidence: (1) well-established genes with functional validation in milk production traits (e.g., *DGAT1*, *ABCG2*, *MGST1*), for which we discuss known mechanisms and the novel THI-dependent patterns observed in this study; and (2) hypothetical candidates with limited or no prior evidence linking them to heat stress (e.g., *CPSF1*, *VPS28*, *PPP1R16A*, *ZNF250*, *GRID2*, *KCNC2*), for which we present the association cautiously and emphasize the need for functional validation. Among these, nine representative genes—*DGAT1*, *CPSF1*, *ABCG2*, *MGST1*, *VPS28*, *PPP1R16A*, *ZNF250*, *GRID2*, and *LOC787350*—were highlighted for further discussion due to their previously reported roles in milk production or stress-related pathways. However, it should be emphasized that their biological roles in heat stress regulation are inferred from known functions and require experimental validation.

*DGAT1* (well-established gene) is located on BTA14 and encodes a key enzyme in triglyceride synthesis [30,31]. In a conventional main-effect GWAS, the *p*-value for a locus near this gene was 1.19 × 10^−68^, confirming its strong main effect; the THI-GWAS further revealed that its effect gradually weakens as THI increases (dynamic test *p* = 4.29 × 10^−86^). These findings are consistent with previous GWAS studies in Chinese Holstein populations that also identified *DGAT1* as a major gene for milk production traits [32]. Notably, *DGAT1* has been reported to be regulated by lipid metabolism transcription factors such as *SREBP1* and *PPARγ*, which are known to respond to nutritional and environmental cues; however, whether heat stress directly modulates *DGAT1* expression through these pathways requires experimental verification.

*CPSF1* (hypothetical candidate) is involved in mRNA 3′-end processing, but its specific role in heat stress responses warrants further investigation. *ABCG2* encodes an ABC transporter involved in the transmembrane transport of milk components [33]; its interaction with THI may be related to heat-induced physiological changes, though the underlying mechanism remains unclear. *MGST1* (well-established gene) participates in lipid metabolism and oxidative stress defense and has been reported to be strongly associated with milk fat percentage [34]; its THI-dependent effect suggests a potential role in coordinating these processes under heat stress, but direct functional evidence is currently lacking. The same region also harbors *EPS8*, previously associated with milk production traits [18,19], suggesting this locus may contain multiple genes affecting milk fat synthesis. *VPS28* (hypothetical candidate) is a component of the *ESCRT-I* complex involved in vesicular transport; its association with milk fat percentage is intriguing but requires functional confirmation. *PPP1R16A* (hypothetical candidate) encodes a regulatory subunit of protein phosphatase 1; no prior evidence links this gene to heat stress, and its role requires investigation.

Sparse reports candidate genes: This study also identified six candidate genes that have been rarely reported in studies on heat stress or THI interactions in dairy cows (*ZNF250*, *GRID2*, *LOC112449570*, *LOC112449590*, *LOC101903754*, and *LOC787350*). Additionally, *KCNC2* on BTA5 was also rarely reported in the literature; however, based on its significant interaction signals across all three environmental dimensions (THI, temperature, and humidity), it met the statistical criteria for a core candidate gene. The functions of all candidate genes identified in this study require validation through subsequent molecular biology experiments and independent replication studies.

### 4.4. Significance of Temperature and Humidity Sensitivity Analysis

To assess the robustness of the THI-GWAS and explore the relative roles of temperature and humidity, we performed separate GWAS using each factor as the environmental variable, aiming to validate the THI findings and investigate whether the composite THI index may obscure stressor-specific genetic signals.

The temperature-interaction GWAS showed substantial overlap with the THI-GWAS, particularly on BTA14. Given the strong correlation between temperature and THI (r = 0.99), this concordance suggests that temperature may be the predominant driver of the THI-dependent effects in these shared regions, supporting the validity of THI as a practical heat stress metric for these loci.

In contrast, the humidity-interaction GWAS revealed a more nuanced pattern. While it shared several loci with the THI-GWAS, it also detected humidity-specific signals (e.g., on BTA5 and BTA16) that were not significant in either THI or temperature analyses. This raises the possibility that humidity may exert independent effects on certain traits or interact with genetic variants in ways partially masked by a composite index, potentially through distinct physiological pathways such as impaired respiratory heat dissipation. However, given the correlations among the three environmental variables, we cannot definitively attribute these signals to humidity independently of temperature based on the current data.

Collectively, these analyses suggest that while THI-based GWAS provides an integrative measure for detecting major environment-dependent loci, separate temperature and humidity analyses offer complementary insights—temperature analysis confirms the robustness of primary signals, whereas humidity analysis may uncover additional associations potentially related to moisture stress. These sensitivity analyses are not intended to replace THI models but to provide a more granular understanding of environmental components contributing to genetic interactions, which may, after further validation, help refine environmental indices for genetic evaluations in specific regions. Distinguishing the independent contributions of temperature and humidity will require further conditional analyses or independent validation populations.

### 4.5. Limitations and Future Research

This study has the following limitations. First, meteorological data were obtained from regional weather stations rather than on-site farm measurements, which may introduce discrepancies between recorded conditions and the actual barn microclimate experienced by cows. Barn microclimate can differ substantially from regional data due to factors such as ventilation, shading, stocking density, and barn design [16,17]. This mismatch could lead to under- or overestimation of heat stress effects on milk traits and may affect the detection of genotype-environment interaction signals. Although we employed nearest-station matching and inverse-distance-weighted interpolation to minimize spatial discrepancies, these methods cannot fully capture farm-specific or within-barn variations. Future studies incorporating on-farm environmental sensors (e.g., temperature-humidity loggers at cow level) would provide more precise exposure assessment.

It is important to acknowledge that this study only considered same-day THI values, and the delayed (lag) effects of heat stress on milk performance were not evaluated. Previous studies have demonstrated that heat stress can have cumulative and carry-over effects on milk production, which may persist for several days following exposure [35]. Future studies incorporating lagged THI variables may provide a more comprehensive understanding of the temporal dynamics of heat stress responses in dairy cattle.

The current THI threshold (THI ≥ 80 indicating severe heat stress) is primarily based on data from Holstein cattle in Europe and the United States; however, its applicability to Chinese populations remains to be verified. Fourth, candidate genes identified in this study require functional validation through molecular biology experiments, and the findings lack external validation in independent populations, which may lead to false positives or overestimation of effects. Although we employed rigorous multiple-testing correction (Bonferroni based on ~12,280 independent SNPs) and a tiered candidate gene classification strategy to control false-positive rates, these statistical measures cannot substitute for independent replication. Future research should focus on: (1) installing real-time environmental monitoring equipment on-site to construct THI lagged variables for assessing cumulative effects; (2) re-estimating heat stress thresholds applicable to Chinese Holstein cattle; (3) constructing mammary epithelial cell heat stress models around core candidate genes and verifying their function through overexpression/knockdown experiments; and (4) collecting data from independent validation populations for external validation.

We also acknowledge that we did not perform a formal model-order selection analysis (e.g., based on AIC or BIC) specifically for our dataset. Instead, we followed the recommendation of Pool et al. [36] and the empirical evidence from Li et al. [37] in a Chinese Holstein population, both of which support the use of a fourth-order polynomial. Nevertheless, we recognize that dataset-specific comparisons could further strengthen the methodological rigor of future random regression GWAS studies, and we suggest this as a direction for further investigation.

### 4.6. Practical Implications for Dairy Breeding Under Climate Change

The findings of this study have several potential implications for dairy cattle breeding under climate change. First, the identification of SNP × THI interaction effects suggests that genetic evaluation models incorporating environmental gradients may improve genomic prediction accuracy for animals in hot and humid regions, as current evaluations typically ignore G×E interactions. Second, the core candidate genes (e.g., *DGAT1*, *ABCG2*, *MGST1*) represent potential targets for marker-assisted selection for heat tolerance, though they require validation in independent populations before practical application. Third, the trait-specific THI response patterns suggest that breeding strategies should adopt different selection indices for different environments—prioritizing fat and protein stability under mild heat stress, and milk yield maintenance under severe heat waves. Finally, this study demonstrates that random regression GWAS is a valuable tool for dissecting environmental adaptability in complex traits, an approach extendable to other livestock species and environmental stressors to support climate-resilient breeding.

## 5. Conclusions

Based on records of Chinese Holstein dairy cows, meteorological data, and SNP chip data, this study systematically evaluated the effects of THI on milk production traits and the associated gene-environment interactions. Phenotypic analysis indicated that as THI increased, milk yield, milk fat percentage, and milk protein percentage generally decreased, although the response patterns varied. A random regression GWAS based on continuous THI identified 149 significant SNP × THI interactions, primarily distributed across BTA5, BTA6, BTA14 and BTA20. Conventional main-effect GWAS detected 94 significant SNPs, whereas THI-GWAS identified 149 significant associations. Overlap analysis showed that 90.9% and 83.7% of the conventional main-effect SNPs overlapped with THI-GWAS loci for milk fat percentage and milk protein percentage, respectively, while no overlap was observed for milk yield. Further annotation identified 52 candidate genes, of which 50 core candidate genes were supported in both the temperature interaction GWAS and the humidity interaction GWAS. Representative core genes include *DGAT1*, *CPSF1*, *ABCG2*, *MGST1*, *VPS28*, *PPP1R16A*, *ZNF250*, *GRID2*, and *LOC787350*; one potential candidate gene (*KCNS2*) and one suggestive candidate gene (*LOC112449570*) require further investigation. In summary, incorporating THI as a continuous environmental variable into a random regression GWAS enables the detection of environment-dependent genetic mechanisms that may not be detected in conventional main-effect GWAS. Additionally, separate temperature- and humidity-based sensitivity analyses can provide complementary information, potentially revealing loci specifically associated with temperature or moisture stress, although these findings require further validation. Together, these approaches may help elucidate the genetic basis of milk production traits under varying thermal environments.

## Figures and Tables

**Table 1:** Descriptive Statistics of Milk Production Traits.

Trait	No. of Cows	No. of Records	Mean	SD	Min	Max
Milk yield (kg)	7240	63,334	32.11	7.32	5.00	71.50
Milk fat percentage (%)	7240	63,334	3.68	0.75	1.40	6.20
Milk protein percentage (%)	7240	63,334	3.11	0.29	2.00	5.00

**Table 2:** Significant QTL regions and representative lead SNPs identified by THI-interaction GWAS for milk production traits in Chinese Holstein cattle.

Trait	Chr	Start Position	End Position	Lead SNP	Min *p*-Value	Number of Significant SNP-Trait Associations	Candidate Gene
MY	14	1	1,109,870	UFL-rs134432442	2.86 × 10^−10^	5	*DGAT1*, *CPSF1*
FP	14	1	4,959,231	ARS-BFGL-NGS-4939	4.29 × 10^−86^	75	*DGAT1*, *CPSF1*, *LOC787350*
	5	92,561,021	94,669,217	BovineHD0500026662	6.57 × 10^−11^	9	*MGST1*, *EPS8*, *SLC15A5*
	14	5,156,988	6,156,988	BovineHD1400001789	3.32 × 10^−6^	1	*LOC112449570*
	16	7,586,136	8,586,136	ARS-BFGL-NGS-109702	1.08 × 10^−6^	1	*LOC101903754*
PP	5	4,017,412	5,017,412	HapMap44984-BTA-72893	1.29 × 10^−6^	1	*KCNC2*
	6	30,567,757	31,567,757	HapMap42776-BTA-105028	7.54 × 10^−7^	1	*GRID2*
	6	35,686,105	37,472,388	BovineHD0600010569	7.33 × 10^−19^	24	*PKD2*
	14	1	1,822,326	UFL-rs134432442	1.26 × 10^−16^	11	*DGAT1*, *CPSF1*
	14	65,295,499	66,295,499	BovineHD1400018987	3.13 × 10^−6^	1	*KCNS2*
	20	30,728,123	34,309,571	UA-IFASA-7069	1.38 × 10^−17^	18	*FRMD4B*, *FAM19A1*
	20	36,400,514	37,400,514	BovineHD2000010588	1.22 × 10^−7^	1	*WDR70*
	20	39,396,176	40,396,176	BovineHD2000011414	1.29 × 10^−6^	1	*ADAMTS12*

## Data Availability

The original contributions presented in this study are included in the article/Supplementary Materials. Further inquiries can be directed to the corresponding authors.

## Supplementary Materials

The following supporting information can be downloaded at: https://www.mdpi.com/article/10.3390/ani16152379/s1, Table S1. Descriptive statistics of milk yield in Chinese Holstein cattle across THI levels. Table S2. Descriptive statistics of milk fat percentage in Chinese Holstein cattle across THI levels. Table S3. Descriptive statistics of milk protein percentage in Chinese Holstein cattle across THI levels. Table S4. Genome-wide significant SNP × THI interaction loci identified by THI-interaction GWAS. Table S5. QTL regions merged from genome-wide significant SNP×THI interaction loci. Table S6. Key candidate genes identified by THI-interaction GWAS and supporting literature. Table S7. Genome-wide significant SNP × temperature interaction loci identified by temperature-interaction GWAS. Table S8. Genome-wide significant SNP × relative humidity interaction loci identified by relative humidity-interaction GWAS. Table S9. Genome-wide significant loci identified by conventional main-effect GWAS and their overlap with THI-interaction GWAS. Table S10. Summary of significant loci and overlaps among THI-, temperature-, relative humidity-interaction GWAS and conventional GWAS. Figure S1. Trends in daily maximum and minimum temperatures in Beijing, Shanghai and Shandong from 2001 to 2023. Note: Blue, red and orange represent Beijing, Shanghai and Shandong respectively. The solid line represents the highest temperature of the day, and the dotted line indicates the lowest temperature of the day.
