# Supplementary material for: Effects of the Temperature–Humidity Index on Milk Production Traits and Gene–Environment Interactions in Chinese Holstein Cows
Source: Animals (Basel). 2026 Aug 3;16(15):2379. doi: 10.3390/ani16152379 (PMC13464799; doi:10.3390/ani16152379)
Supplement: Supplementary file 1 [file animals-16-02379-s001.zip › animals-4446063-supplementary.pdf]

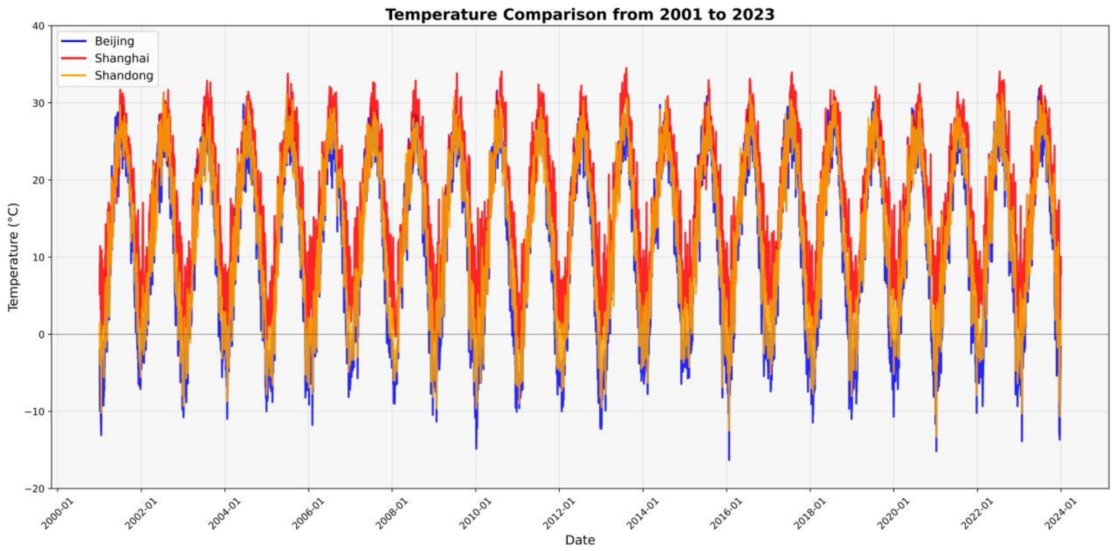

Figure S1. Trends in daily maximum and minimum temperatures in Beijing, Shanghai and Shandong from 2001 to 2023.

Note: Blue, red and orange represent Beijing, Shanghai and Shandong respectively. The solid line represents the highest temperature of the day, and the dotted line indicates the lowest temperature of the day.

Table S1. Descriptive statistics of milk yield in Chinese Holstein cattle across THI levels

THI Level	THI	Sample size	Mean milk yield (kg)	Standard deviation	Standard error.
	24	92	30.072	6.521	0.68
	25	45	32.8	5.723	0.853
	26	111	31.383	6.375	0.605
	27	75	28.693	6.01	0.694
	28	149	29.958	5.931	0.486
	29	128	33.108	6.507	0.575
	30	455	32.554	7.265	0.341
	31	592	32.568	7.091	0.291
	32	618	33.592	7.093	0.285
No heat stress	33	925	33.202	7.139	0.235
	34	1320	33.603	7.1	0.195
	35	1404	32.895	6.927	0.185
	36	1706	32.674	6.783	0.164
	37	1276	32.516	7.255	0.203
	38	1572	33.853	7.366	0.186
	39	1134	32.395	6.955	0.207
	40	943	32.227	7.651	0.249
	41	1421	32.472	7.222	0.192
	42	1349	31.842	6.966	0.19

	43	818	30.881	6.605	0.231
	44	1366	32.539	7.448	0.202
	45	853	31.497	7.223	0.247
	46	741	31.761	7.131	0.262
	47	926	32.224	7.673	0.252
	48	911	32.194	7.503	0.249
	49	1015	32.869	7.284	0.229
	50	1182	32.081	7.209	0.21
	51	1195	31.925	7.641	0.221
	52	1114	31.322	6.316	0.189
	53	1459	33.294	7.336	0.192
	54	1372	31.225	7.133	0.193
	55	945	31.45	6.83	0.222
	56	1704	32.76	6.83	0.165
	57	1038	34.024	7.521	0.233
	58	1083	32.454	7.378	0.224
	59	949	33.619	7.716	0.251
	60	1147	33.724	7.453	0.22
	61	1058	33.184	7.261	0.223
	62	802	32.349	7.632	0.269
	63	962	31.034	6.706	0.216
	64	1454	32.752	6.899	0.181
	65	1382	31.784	7.497	0.202
	66	1208	32.131	7.494	0.216
	67	1345	32.439	7.292	0.199
	68	1603	32.995	7.686	0.192
Mild Heat Stress	69	986	31.981	6.754	0.215
	70	1668	32.391	6.99	0.171
	71	1619	32.571	7.003	0.174
	72	2085	32.25	7.399	0.162
	73	1673	31.517	7.473	0.183
	74	2953	32.376	7.257	0.134
Moderate heat stress	75	1692	30.641	7.271	0.177
	76	1942	31.204	6.699	0.152
	77	704	30.438	7.249	0.273
	78	912	28.66	7.336	0.243
	79	747	31.47	7.47	0.273
	80	487	28.557	7.712	0.349
Severe heat stress	81	382	25.678	7.008	0.359
	82	428	23.598	5.708	0.276
	83	109	25.314	5.636	0.54

Table S2. Descriptive statistics of milk fat percentage in Chinese Holstein cattle across THI levels

THI Level	THI	Sample Size	Mean Milk Fat Percentage (%)	Standard Deviation	Standard Error.
No heat stress	24	92	4.092	0.825	0.086
	25	45	3.517	0.455	0.068
	26	111	4.095	0.713	0.068
	27	75	3.947	0.671	0.078
	28	149	4.063	0.745	0.061
	29	128	3.874	0.733	0.065
	30	455	3.729	0.704	0.033
	31	592	3.85	0.765	0.031
	32	618	3.784	0.742	0.03
	33	925	3.825	0.717	0.024
	34	1320	3.944	0.74	0.02
	35	1404	3.941	0.733	0.02
	36	1706	3.919	0.735	0.018
	37	1276	3.795	0.722	0.02
	38	1572	3.796	0.736	0.019
	39	1134	3.76	0.755	0.022
	40	943	3.812	0.752	0.024
	41	1421	3.781	0.767	0.02
	42	1349	3.816	0.765	0.021
	43	818	3.844	0.82	0.029
	44	1366	3.806	0.737	0.02
	45	853	3.919	0.704	0.024
	46	741	3.84	0.782	0.029
	47	926	3.738	0.831	0.027
	48	911	3.782	0.787	0.026
	49	1015	3.719	0.726	0.023
	50	1182	3.716	0.73	0.021
	51	1195	3.711	0.761	0.022
	52	1114	3.785	0.695	0.021
	53	1459	3.756	0.751	0.02
	54	1372	3.747	0.778	0.021
	55	945	3.704	0.712	0.023
	56	1704	3.788	0.728	0.018
	57	1038	3.687	0.795	0.025
	58	1083	3.722	0.758	0.023
	59	949	3.481	0.699	0.023
	60	1147	3.514	0.746	0.022
	61	1058	3.655	0.72	0.022

	62	802	3.685	0.714	0.025
	63	962	3.661	0.744	0.024
	64	1454	3.577	0.731	0.019
	65	1382	3.556	0.704	0.019
	66	1208	3.665	0.764	0.022
	67	1345	3.602	0.702	0.019
	68	1603	3.578	0.735	0.018
Mild Heat Stress	69	986	3.663	0.691	0.022
	70	1668	3.485	0.697	0.017
	71	1619	3.479	0.682	0.017
	72	2085	3.508	0.729	0.016
	73	1673	3.451	0.719	0.018
	74	2953	3.509	0.694	0.013
Moderate heat stress	75	1692	3.485	0.722	0.018
	76	1942	3.667	0.744	0.017
	77	704	3.63	0.691	0.026
	78	912	3.445	0.701	0.023
	79	747	3.535	0.726	0.027
	80	487	3.606	0.792	0.036
Severe heat stress	81	382	3.413	0.759	0.039
	82	428	3.538	0.694	0.034
	83	109	3.452	0.487	0.047

Table S3. Descriptive statistics of milk protein percentage in Chinese Holstein cattle across THI levels

THI Level	THI	Sample Size	Mean Protein Percentage（%）	Standard Deviation	Standard Error.
	24	92	3.285	0.248	0.026
	25	45	3.153	0.242	0.036
	26	111	3.21	0.323	0.031
	27	75	3.199	0.325	0.038
No heat stress	28	149	3.109	0.258	0.021
	29	128	3.295	0.274	0.024
	30	455	3.196	0.259	0.012
	31	592	3.186	0.261	0.011
	32	618	3.182	0.282	0.011

	33	925	3.203	0.272	0.009
	34	1320	3.203	0.295	0.008
	35	1404	3.201	0.28	0.007
	36	1706	3.197	0.286	0.007
	37	1276	3.199	0.286	0.008
	38	1572	3.191	0.275	0.007
	39	1134	3.201	0.301	0.009
	40	943	3.188	0.308	0.01
	41	1421	3.212	0.294	0.008
	42	1349	3.185	0.302	0.008
	43	818	3.184	0.322	0.011
	44	1366	3.16	0.289	0.008
	45	853	3.146	0.304	0.01
	46	741	3.146	0.314	0.012
	47	926	3.188	0.278	0.009
	48	911	3.134	0.312	0.01
	49	1015	3.193	0.29	0.009
	50	1182	3.142	0.283	0.008
	51	1195	3.167	0.285	0.008
	52	1114	3.113	0.298	0.009
	53	1459	3.134	0.303	0.008
	54	1372	3.151	0.308	0.008
	55	945	3.115	0.311	0.01
	56	1704	3.078	0.309	0.007
	57	1038	3.114	0.26	0.008
	58	1083	3.134	0.3	0.009
	59	949	3.088	0.25	0.008
	60	1147	3.095	0.268	0.008
	61	1058	3.074	0.255	0.008
	62	802	3.191	0.306	0.011
	63	962	3.081	0.289	0.009
	64	1454	3.079	0.261	0.007
	65	1382	3.077	0.269	0.007
	66	1208	3.077	0.285	0.008
	67	1345	3.069	0.263	0.007
	68	1603	3.022	0.266	0.007
	69	986	3.048	0.242	0.008
Mild Heat Stress	70	1668	3.056	0.259	0.006
	71	1619	3.022	0.273	0.007
	72	2085	3.042	0.269	0.006
	73	1673	3.013	0.267	0.007
Moderate heat stress	74	2953	3.007	0.243	0.004
	75	1692	3.018	0.27	0.007
	76	1942	2.999	0.253	0.006

	77	704	3.045	0.276	0.01
	78	912	3.06	0.293	0.01
	79	747	2.99	0.259	0.009
Severe heat stress	80	487	3.055	0.29	0.013
	81	382	2.969	0.297	0.015
	82	428	3.027	0.294	0.014
	83	109	2.955	0.238	0.023

Table S4. Genome-wide significant SNP×THI interaction loci identified by THI-interaction GWAS

Trait	Chr	SNP ID	Physical Location (bp)	P-value	MAF	Nearest Genes	Candidate Genes	QTL Region
MY	14	BovineHD1400000188	403312	1.35E-09	0.237	CPSF1	CPSF1, DGAT1	BTA14_region1
MY	14	ARS-BFGL-NGS-57820	465742	1.04E-09	0.236	CPSF1	CPSF1, DGAT1	BTA14_region1
MY	14	BovineHD1400000206	494621	6.03E-10	0.215	CPSF1	CPSF1, DGAT1	BTA14_region1
MY	14	UFL-rs134432442	550784	2.86E-10	0.214	CPSF1	CPSF1, DGAT1	BTA14_region1
MY	14	ARS-BFGL-NGS-4939	609870	3.30E-10	0.215	DGAT1	DGAT1,CPSF1	BTA14_region1
FP	5	BovineHD0500026533	93061021	1.22E-06	0.193	MGST1	MGST1, EPS8, SLC15A5	BTA5_region2
FP	5	BovineHD0500026557	93124633	3.51E-08	0.485	MGST1	MGST1, EPS8, SLC15A5	BTA5_region2
FP	5	BovineHD0500026635	93413676	2.52E-08	0.129	MGST1	MGST1, EPS8, SLC15A5	BTA5_region2
FP	5	BovineHD0500026655	93503991	6.76E-08	0.423	MGST1	MGST1, EPS8, SLC15A5	BTA5_region2
FP	5	BovineHD0500026662	93515983	6.57E-11	0.317	MGST1	MGST1, EPS8, SLC15A5	BTA5_region2
FP	5	BovineHD0500026668	93524134	4.12E-10	0.48	MGST1	MGST1, EPS8, SLC15A5	BTA5_region2
FP	5	BovineHD0500026682	93566261	4.29E-09	0.429	MGST1	MGST1, EPS8, SLC15A5	BTA5_region2
FP	5	BovineHD0500026721	93679644	1.93E-07	0.494	MGST1	MGST1, EPS8, SLC15A5	BTA5_region2
FP	5	Hapmap41349-BTA-74576	94169217	3.33E-07	0.081			BTA5_region2
FP	14	BovineHD1400024339	146715	2.56E-16	0.468	DGAT1	DGAT1	BTA14_region1
FP	14	BovineHD1400000138	223706	1.02E-14	0.484	DGAT1	DGAT1	BTA14_region1
FP	14	BovineHD1400000143	243959	5.54E-13	0.479	DGAT1	DGAT1	BTA14_region1
FP	14	BovineHD1400000152	255765	3.22E-10	0.448	DGAT1	DGAT1	BTA14_region1
FP	14	Hapmap30381-BTC-005750	279949	2.07E-12	0.299	DGAT1	DGAT1	BTA14_region1
FP	14	Hapmap30383-BTC-005848	305780	1.99E-27	0.354	DGAT1	DGAT1	BTA14_region1
FP	14	BTA-34956-no-rs	330256	9.53E-11	0.474	DGAT1	DGAT1	BTA14_region1

FP	14	BovineHD1400000187	399818	1.23E-08	0.464	DGAT1	DGAT1	BTA14_region1
FP	14	BovineHD1400000188	403312	1.11E-61	0.237	DGAT1/ CPSF1	DGAT1/CPSF1	BTA14_region1
FP	14	ARS-BFGL-NGS-57820	465742	3.53E-60	0.236	DGAT1/ CPSF1	DGAT1/CPSF1	BTA14_region1
FP	14	ARS-BFGL-NGS-34135	490055	1.76E-24	0.385	DGAT1/ CPSF1	DGAT1/CPSF1	BTA14_region1
FP	14	BovineHD1400000206	494621	5.66E-69	0.215	DGAT1/ CPSF1	DGAT1/CPSF1	BTA14_region1
FP	14	ARS-BFGL-NGS-94706	511247	2.20E-25	0.398	DGAT1/ CPSF1	DGAT1/CPSF1	BTA14_region1
FP	14	UFL-rs134432442	550784	1.18E-84	0.214	DGAT1/ CPSF1	DGAT1/CPSF1	BTA14_region1
FP	14	ARS-BFGL-NGS-4939	609870	4.29E-86	0.215	DGAT1	DGAT1	BTA14_region1
FP	14	BovineHD1400000243	676575	1.02E-28	0.395	DGAT1	DGAT1	BTA14_region1
FP	14	BovineHD1400000246	688317	1.00E-27	0.4	DGAT1	DGAT1	BTA14_region1
FP	14	BovineHD1400000249	700497	9.50E-28	0.4	DGAT1	DGAT1	BTA14_region1
FP	14	Hapmap52798-ss465264 55	731230	4.23E-18	0.443	DGAT1	DGAT1	BTA14_region1
FP	14	ARS-BFGL-NGS-71749	762253	1.86E-08	0.185			BTA14_region1
FP	14	BovineHD1400000262	775260	1.05E-49	0.248	DGAT1	DGAT1	BTA14_region1
FP	14	UA-IFASA-6878	810863	1.21E-18	0.414	DGAT1	DGAT1	BTA14_region1
FP	14	ARS-BFGL-NGS-107379	862483	3.96E-53	0.263	DGAT1	DGAT1	BTA14_region1
FP	14	BovineHD1400000286	876529	2.65E-52	0.257	DGAT1	DGAT1	BTA14_region1
FP	14	BovineHD1400000288	891340	9.32E-15	0.366	DGAT1	DGAT1	BTA14_region1
FP	14	ARS-BFGL-NGS-18365	924727	1.76E-11	0.32	DGAT1	DGAT1	BTA14_region1
FP	14	Hapmap30922-BTC-0020 21	946190	3.25E-09	0.294	DGAT1	DGAT1	BTA14_region1
FP	14	BovineHD1400000301	959635	1.37E-08	0.353	DGAT1	DGAT1	BTA14_region1
FP	14	BovineHD1400000305	973229	3.98E-08	0.332	DGAT1	DGAT1	BTA14_region1
FP	14	Hapmap25384-BTC-0019 97	1022847	9.45E-12	0.466	DGAT1	DGAT1	BTA14_region1
FP	14	Hapmap24715-BTC-0019 73	1044777	2.48E-12	0.461	DGAT1	DGAT1	BTA14_region1
FP	14	BTA-35941-no-rs	1082140	6.99E-15	0.435	DGAT1	DGAT1	BTA14_region1
FP	14	ARS-BFGL-NGS-101653	1119139	1.63E-08	0.216	DGAT1	DGAT1	BTA14_region1
FP	14	BovineHD1400000341	1142313	4.42E-16	0.429	DGAT1	DGAT1	BTA14_region1
FP	14	ARS-BFGL-NGS-26520	1184910	6.60E-13	0.305	DGAT1	DGAT1	BTA14_region1
FP	14	BovineHD4100010534	1197094	3.57E-16	0.429	DGAT1	DGAT1	BTA14_region1
FP	14	Hapmap30086-BTC-0020 66	1322326	3.29E-19	0.474	DGAT1	DGAT1	BTA14_region1
FP	14	BovineHD1400000397	1344741	1.39E-14	0.442	DGAT1	DGAT1	BTA14_region1
FP	14	Hapmap30646-BTC-0020 54	1351419	3.90E-13	0.394	DGAT1	DGAT1	BTA14_region1

FP	14	Hapmap25486-BTC-072553	1567838	1.06E-07	0.259	DGAT1	DGAT1	BTA14_region1
FP	14	BovineHD1400000447	1610072	1.69E-08	0.486	DGAT1	DGAT1	BTA14_region1
FP	14	ARS-BFGL-NGS-103064	1620025	1.03E-08	0.486	DGAT1	DGAT1	BTA14_region1
FP	14	BovineHD1400000453	1657677	4.64E-10	0.353	DGAT1	DGAT1	BTA14_region1
FP	14	ARS-BFGL-NGS-22866	1681783	3.62E-08	0.369	DGAT1	DGAT1	BTA14_region1
FP	14	BovineHD1400000467	1747091	5.53E-19	0.428	DGAT1	DGAT1	BTA14_region1
FP	14	ARS-BFGL-NGS-18858	1758505	1.33E-19	0.426	DGAT1	DGAT1	BTA14_region1
FP	14	BovineHD1400000480	1785051	1.57E-08	0.474	DGAT1	DGAT1	BTA14_region1
FP	14	ARS-BFGL-NGS-59769	1854457	4.62E-08	0.463	DGAT1	DGAT1	BTA14_region1
FP	14	BovineHD1400024350	1896438	1.36E-10	0.35	DGAT1	DGAT1	BTA14_region1
FP	14	Hapmap36620-SCAFFOLD50018_7571	2142001	3.40E-13	0.461	DGAT1	DGAT1	BTA14_region1
FP	14	BovineHD1400000616	2216794	2.12E-24	0.483	DGAT1	DGAT1	BTA14_region1
FP	14	BovineHD1400000654	2309238	9.14E-08	0.281	DGAT1	DGAT1	BTA14_region1
FP	14	ARS-BFGL-NGS-74378	2616322	3.04E-09	0.326	DGAT1	DGAT1	BTA14_region1
FP	14	ARS-BFGL-NGS-117542	2640274	9.43E-07	0.232	DGAT1	DGAT1	BTA14_region1
FP	14	BovineHD1400000755	2719625	2.19E-06	0.28	DGAT1	DGAT1	BTA14_region1
FP	14	BovineHD1400000788	2840340	8.32E-09	0.446	DGAT1	DGAT1	BTA14_region1
FP	14	BovineHD1400000792	2867641	7.33E-08	0.383	DGAT1	DGAT1	BTA14_region1
FP	14	UA-IFASA-9288	2929132	3.62E-14	0.308	DGAT1	DGAT1	BTA14_region1
FP	14	Hapmap32970-BTC-064990	3015800	1.58E-07	0.385	DGAT1	DGAT1	BTA14_region1
FP	14	Hapmap24986-BTC-065021	3040885	2.81E-07	0.384	DGAT1	DGAT1	BTA14_region1
FP	14	BovineHD1400000870	3106715	2.24E-09	0.493	DGAT1	DGAT1	BTA14_region1
FP	14	Hapmap26072-BTC-065132	3120000	2.79E-07	0.308	DGAT1	DGAT1	BTA14_region1
FP	14	Hapmap26527-BTC-005059	3147254	1.22E-07	0.312	DGAT1	DGAT1	BTA14_region1
FP	14	ARS-BFGL-NGS-56327	3308936	4.94E-08	0.385	DGAT1	DGAT1	BTA14_region1
FP	14	ARS-BFGL-NGS-100480	3336128	2.73E-14	0.454	DGAT1	DGAT1	BTA14_region1
FP	14	BovineHD1400000977	3385942	8.29E-07	0.379	DGAT1	DGAT1	BTA14_region1
FP	14	BovineHD1400000999	3427682	9.35E-07	0.411	DGAT1	DGAT1	BTA14_region1
FP	14	UA-IFASA-5306	3439565	2.49E-15	0.32	DGAT1	DGAT1	BTA14_region1
FP	14	Hapmap27703-BTC-053907	3554540	4.74E-08	0.351	DGAT1	DGAT1	BTA14_region1
FP	14	BovineHD4100010659	3730901	7.41E-07	0.303	DGAT1	DGAT1	BTA14_region1
FP	14	Hapmap23302-BTC-052123	3819126	2.92E-06	0.294	DGAT1	DGAT1	BTA14_region1
FP	14	ARS-BFGL-NGS-3571	4188954	6.58E-07	0.439	DGAT1	DGAT1	BTA14_region1
FP	14	BovineHD4100010689	4257911	2.11E-06	0.496	DGAT1	DGAT1	BTA14_region1
FP	14	ARS-BFGL-NGS-115947	4459231	1.63E-07	0.42	DGAT1	DGAT1	BTA14_region1
FP	14	BovineHD1400001789	5656988	3.32E-06	0.464	LOC1124	LOC112449570	BTA14_region2

						49570		
FP	16	ARS-BFGL-NGS-109702	8086136	1.08E-06	0.044	LOC1019 03754	LOC101903754	BTA16_region1
FP	14	BovineHD1400024034	52575	2.02E-08	0.183	LOC7873 50	LOC787350	BTA14_region1
PP	5	Hapmap44984-BTA-728 93	4517412	1.29E-06	0.366	KCNC2	KCNC2	BTA5_region1
PP	6	Hapmap42776-BTA-105 028	31067757	7.54E-07	0.011	GRID2	GRID2	BTA6_region1
PP	6	BovineHD4100004508	36186105	2.06E-15	0.029	PKD2	PKD2	BTA6_region2
PP	6	Hapmap24324-BTC-0624 49	36205216	1.87E-15	0.029	PKD2	PKD2	BTA6_region2
PP	6	BovineHD4100004514	36218002	1.52E-15	0.028	PKD2	PKD2	BTA6_region2
PP	6	BovineHD4100004517	36241100	1.64E-12	0.034	PKD2	PKD2	BTA6_region2
PP	6	BovineHD0600010428	36251003	3.45E-15	0.029	PKD2	PKD2	BTA6_region2
PP	6	BovineHD4100004526	36314103	5.70E-14	0.03	PKD2	PKD2	BTA6_region2
PP	6	BovineHD0600010444	36326417	1.23E-15	0.029	PKD2	PKD2	BTA6_region2
PP	6	BovineHD0600010445	36330445	2.07E-07	0.032	PKD2	PKD2	BTA6_region2
PP	6	BovineHD4100004528	36332467	1.60E-13	0.031	PKD2	PKD2	BTA6_region2
PP	6	BovineHD4100004529	36334803	1.64E-15	0.029	PKD2	PKD2	BTA6_region2
PP	6	BovineHD4100004530	36342054	8.38E-16	0.029	PKD2	PKD2	BTA6_region2
PP	6	BovineHD4100004531	36364608	1.23E-15	0.03	PKD2	PKD2	BTA6_region2
PP	6	BovineHD0600010555	36592235	2.01E-10	0.087	PKD2	PKD2	BTA6_region2
PP	6	BTA-121739-no-rs	36635943	6.45E-11	0.113	PKD2	PKD2	BTA6_region2
PP	6	BovineHD0600010569	36657367	7.33E-19	0.048	PKD2	PKD2	BTA6_region2
PP	6	BovineHD0600010605	36855289	2.54E-08	0.233	PKD2	PKD2	BTA6_region2
PP	6	Hapmap26259-BTC-0335 26	36892307	3.15E-07	0.262	PKD2	PKD2	BTA6_region2
PP	6	BovineHD4100004553	36915121	2.46E-06	0.289	PKD2	PKD2	BTA6_region2
PP	6	BovineHD0600010624	36942473	5.47E-16	0.114	PKD2	PKD2	BTA6_region2
PP	6	BovineHD0600010625	36943641	1.98E-10	0.18	PKD2	PKD2	BTA6_region2
PP	6	BovineHD4100004557	36944790	1.48E-10	0.182	PKD2	PKD2	BTA6_region2
PP	6	BovineHD4100004558	36956973	4.16E-15	0.058	PKD2	PKD2	BTA6_region2
PP	6	BovineHD0600010630	36970336	3.31E-17	0.054	PKD2	PKD2	BTA6_region2
PP	6	BovineHD4100004560	36972388	7.01E-17	0.055	PKD2	PKD2	BTA6_region2
PP	14	BovineHD1400000188	403312	5.72E-12	0.237	DGAT1/ CPSF1	DGAT1/CPSF1	BTA14_region1
PP	14	ARS-BFGL-NGS-57820	465742	3.06E-11	0.236	DGAT1/ CPSF1	DGAT1/CPSF1	BTA14_region1
PP	14	ARS-BFGL-NGS-34135	490055	7.62E-07	0.385	DGAT1/ CPSF1	DGAT1/CPSF1	BTA14_region1
PP	14	BovineHD1400000206	494621	4.13E-14	0.215	DGAT1/ CPSF1	DGAT1/CPSF1	BTA14_region1
PP	14	ARS-BFGL-NGS-94706	511247	3.57E-07	0.398	DGAT1/	DGAT1/CPSF1	BTA14_region1

PP	14	UFL-rs134432442	550784	1.26E-16	0.214	CPSF1 DGAT1/ CPSF1	DGAT1/CPSF1	BTA14_region1
PP	14	ARS-BFGL-NGS-4939	609870	3.03E-16	0.215	DGAT1	DGAT1	BTA14_region1
PP	14	BovineHD1400000262	775260	2.10E-09	0.248	DGAT1	DGAT1	BTA14_region1
PP	14	ARS-BFGL-NGS-107379	862483	2.26E-09	0.263	DGAT1	DGAT1	BTA14_region1
PP	14	BovineHD1400000286	876529	1.18E-09	0.257	DGAT1	DGAT1	BTA14_region1
PP	14	Hapmap30086-BTC-002066	1322326	2.91E-06	0.474	DGAT1	DGAT1	BTA14_region1
PP	14	BovineHD1400018987	65795499	3.13E-06	0.091	KCNS2	KCNS2	BTA14_region3
PP	20	BovineHD2000009025	31228123	2.46E-07	0.222	FRMD4B	FRMD4B	BTA20_region1
PP	20	BovineHD2000009068	31423773	4.69E-07	0.425	FRMD4B	FRMD4B	BTA20_region1
PP	20	BovineHD2000009105	31591442	5.92E-08	0.173	FRMD4B	FRMD4B	BTA20_region1
PP	20	BovineHD2000009188	31890580	1.98E-10	0.493	FRMD4B	FRMD4B	BTA20_region1
PP	20	UA-IFASA-7069	31912365	1.38E-17	0.344	FRMD4B	FRMD4B	BTA20_region1
PP	20	BovineHD2000009204	31963910	1.24E-10	0.494	FRMD4B	FRMD4B	BTA20_region1
PP	20	BovineHD2000009215	31980361	1.93E-10	0.494	FRMD4B	FRMD4B	BTA20_region1
PP	20	ARS-BFGL-NGS-118998	32009781	3.30E-16	0.351	FRMD4B	FRMD4B	BTA20_region1
PP	20	BovineHD2000009226	32025240	3.40E-17	0.349	FRMD4B	FRMD4B	BTA20_region1
PP	20	BovineHD2000009234	32035193	5.17E-17	0.348	FRMD4B	FRMD4B	BTA20_region1
PP	20	BovineHD2000009251	32077537	6.15E-16	0.351	FRMD4B	FRMD4B	BTA20_region1
PP	20	BovineHD2000009262	32127333	1.14E-08	0.481	FRMD4B	FRMD4B	BTA20_region1
PP	20	BovineHD2000009307	32372127	5.63E-08	0.293	FRMD4B	FRMD4B	BTA20_region1
PP	20	BovineHD2000009361	32694119	1.49E-07	0.414	FAM19A1	FAM19A1	BTA20_region1
PP	20	BovineHD2000009459	33024411	1.81E-11	0.366	FAM19A1	FAM19A1	BTA20_region1
PP	20	ARS-BFGL-NGS-26909	33537716	4.17E-10	0.219		FAM19A1	BTA20_region1
PP	20	BovineHD2000009668	33541612	3.52E-06	0.273		FAM19A1	BTA20_region1
PP	20	BovineHD2000009742	33809571	3.88E-07	0.139		FAM19A1	BTA20_region1
PP	20	BovineHD2000010588	36900514	1.22E-07	0.162	WDR70	WDR70	BTA20_region2
PP	20	BovineHD2000011414	39896176	1.29E-06	0.144	ADAMTS12	ADAMTS12	BTA20_region3

Note: This table lists all SNPs that reached the genome-wide significance threshold, i.e., “significant SNPs”; the SNP with the smallest P-value within each QTL region is designated as the lead SNP for that region (see Appendix Table 5).

Table S5. QTL regions merged from genome-wide significant SNP×THI interaction loci

Trait	Chr	Start position	End position	lead SNP	lead SNP position(bp)	Minimum p-value	Number of significant SNPs	Candidate genes	Sharing of multiple traits
MY	14	1	1109870	UFL-rs134432442	550784	2.86e-10	5	CPSF1, DGAT1	Yes
FP	5	92561021	94669217	BovineHD0500026662	93515983	6.57e-11	9	MGST1, EPS8, SLC15A5	No
FP	14	1	4959231	ARS-BFGL-NGS-4939	609870	4.29e-86	75	DGAT1, CPSF1, LOC787350	Yes
FP	14	5156988	6156988	BovineHD1400001789	5656988	3.32e-06	1	LOC112449570	No
FP	16	7586136	8586136	ARS-BFGL-NGS-109702	8086136	1.08e-06	1	LOC101903754	No
PP	5	4017412	5017412	Hapmap44984-BTA-72893	4517412	1.29e-06	1	KCNC2	No
PP	6	30567757	31567757	Hapmap42776-BTA-105028	31067757	7.54e-07	1	GRID2	No
PP	6	35686105	37472388	BovineHD0600010569	36657367	7.33e-19	24	PKD2	No
PP	14	1	1822326	UFL-rs134432442	550784	1.26e-16	11	DGAT1, CPSF1	Yes
PP	14	65295499	66295499	BovineHD1400018987	65795499	3.13e-06	1	KCNS2	No
PP	20	30728123	34309571	UA-IFASA-7069	31912365	1.38e-17	18	FRMD4B,FAM19A1	No
PP	20	36400514	37400514	BovineHD2000010588	36900514	1.22e-07	1	WDR70	No
PP	20	39396176	40396176	BovineHD2000011414	39896176	1.29e-06	1	ADAMTS12	No

Note: The rules for merging QTL regions are as follows: Significant SNPs on the same chromosome are merged if their physical distance is less than 1 Mb; after merging, the QTL boundaries are defined by extending 500 kb in each direction. For single-SNP regions, the QTL boundaries are defined by extending 500 kb upstream and downstream from the SNP's position. The table lists each QTL region and its representative SNP (the significant SNP with the smallest P-value within the region). BTA14_region1 (1--4,959,231 bp) showed 75, 11, and 5 significant SNP-trait associations in FP, PP, and MY, respectively, with partial SNP overlap among the traits (see Supplementary Table 4 for details); the table lists the representative SNPs separately for FP and PP. If the QTL region boundaries extend beyond the physical chromosome range after a 500-kb expansion, the starting boundary is set to 1 bp.

Table S6. Key candidate genes identified by THI-interaction GWAS and supporting literature

No.	Gene	Trait	Chr	QTL regions	Representative SNP	THI-GWAS P-value	Temperature GWAS P-value	Humidity GWAS P-value	T Support	RH Support	Candidate Gene Level	Literature Evidence	Reports on Heat Stress/THI Interactions
1	ZNF250	FP	14	BTA14_region1	BovineHD1400000188	1.11E-61	1.57E-61	3.42E-61	Yes	Yes	Core Candidate Genes	This study is the first to propose a link between this and its interaction with heat stress/THI	Sparse reports
2	C14H8orf33	FP	14	BTA14_region1	ARS-BFGL-NGS-4939	4.29E-86	8.89E-87	2.18E-86	Yes	Yes	Core Candidate Genes	This study is the first to identify this gene in the context of heat stress/THI interactions in dairy cattle.	Not reported (new candidate)
3	ARHGAP39	FP	14	BTA14_region1	ARS-BFGL-NGS-4939	4.29E-86	8.89E-87	2.18E-86	Yes	Yes	Core Candidate Genes	This study is the first to identify this gene in the context of heat stress/THI interactions in dairy cattle.	Not reported (new candidate)
4	PPP1R16A	FP	14	BTA14_region1	ARS-BFGL-NGS-4939	4.29E-86	8.89E-87	2.18E-86	Yes	Yes	Core Candidate Genes	Liu, 2014; Buitenhuis et al., 2014; Prakapenka et al., 2024	Reported
5	CYHR1	FP	14	BTA14_region1	BovineHD1400000188	1.11E-61	1.57E-61	3.42E-61	Yes	Yes	Core Candidate Genes	Buitenhuis et al., 2014; Prakapenka et al., 2024	Reported
6	VPS28	FP	14	BTA14_region1	BovineHD1400000188	1.11E-61	1.57E-61	3.42E-61	Yes	Yes	Core Candidate Genes	Liu, 2014	Reported
7	CPSF1	FP, MY, PP	14	BTA14_region1	UFL-rs134432442	1.26E-16	3.81E-17	7.03E-17	Yes	Yes	Core Candidate Genes	Liu, 2014; Buitenhuis et al., 2014; Ariyarathne et al., 2021; Chen	Reported

												et al., 2025	
8	DGAT1	FP, MY, PP	14	BTA14_region1	ARS-BFGL-NGS-4939	4.29E-86	8.89E-87	2.18E-86	Yes	Yes	Core Candidate Genes	Grisart et al., 2002; Grisart et al., 2004; Jiang et al., 2010	Reported
9	MROH1	FP	14	BTA14_region1	BovineHD1400000243	1.02E-28	1.47E-29	1.64E-29	Yes	Yes	Core Candidate Genes	Capomaccio et al., 2015; Nayeri et al., 2016	Reported
10	MAF1	FP	14	BTA14_region1	Hapmap52798-ss46526455	4.23E-18	1.76E-18	6.43E-19	Yes	Yes	Core Candidate Genes	Jiang et al.2014	Reported
11	SMPD5	FP	14	BTA14_region1	ARS-BFGL-NGS-4939	4.29E-86	8.89E-87	2.18E-86	Yes	Yes	Core Candidate Genes	This study is the first to identify this gene in the context of heat stress/THI interactions in dairy cattle.	Not reported (new candidate)
12	LOC112449562	FP	14	BTA14_region1	BovineHD1400000188	1.11E-61	1.57E-61	3.42E-61	Yes	Yes	Core Candidate Genes	This study is the first to identify this gene in the context of heat stress/THI interactions in dairy cattle.	Not reported (new candidate)
13	PLEC	FP	14	BTA14_region1	ARS-BFGL-NGS-4939	4.29E-86	8.89E-87	2.18E-86	Yes	Yes	Core Candidate Genes	This study is the first to identify this gene in the context of heat stress/THI interactions in dairy cattle.	Not reported (new candidate)
14	EPPK1	FP	14	BTA14_region1	ARS-BFGL-NGS-4939	4.29E-86	8.89E-87	2.18E-86	Yes	Yes	Core Candidate Genes	This study is the first to identify this gene in the context of heat stress/THI interactions in dairy cattle.	Not reported (new candidate)

15	IQANK1	FP	14	BTA14_region1	ARS-BFGL-NGS-4939	4.29E-86	8.89E-87	2.18E-86	Yes	Yes	Core Candidate Genes	This study is the first to identify this gene in the context of heat stress/THI interactions in dairy cattle.	Not reported (new candidate)
16	MAPK15	FP	14	BTA14_region1	ARS-BFGL-NGS-4939	4.29E-86	8.89E-87	2.18E-86	Yes	Yes	Core Candidate Genes	This study is the first to identify this gene in the context of heat stress/THI interactions in dairy cattle.	Not reported (new candidate)
17	EEF1D	FP	14	BTA14_region1	BovineHD1400000262	1.05E-49	8.66E-50	5.23E-50	Yes	Yes	Core Candidate Genes	Liu, 2014	Reported
18	GSDMD	FP	14	BTA14_region1	ARS-BFGL-NGS-4939	4.29E-86	8.89E-87	2.18E-86	Yes	Yes	Core Candidate Genes	This study is the first to identify this gene in the context of heat stress/THI interactions in dairy cattle.	Not reported (new candidate)
19	ZC3H3	FP	14	BTA14_region1	ARS-BFGL-NGS-4939	4.29E-86	8.89E-87	2.18E-86	Yes	Yes	Core Candidate Genes	This study is the first to identify this gene in the context of heat stress/THI interactions in dairy cattle.	Not reported (new candidate)
20	ZNF696	FP	14	BTA14_region1	ARS-BFGL-NGS-4939	4.29E-86	8.89E-87	2.18E-86	Yes	Yes	Core Candidate Genes	This study is the first to identify this gene in the context of heat stress/THI interactions in dairy cattle.	Not reported (new candidate)
21	LOC	FP	14	BTA14_r	BovineHD1400000	1.11E-61	1.57E-61	3.42E-61	Yes	Yes	Core	This study is	Sparse reports

	1124 4959 0			egion1	188						Candidate Genes	the first to propose a link between this and its interaction with heat stress/THI	
22	LOC 1008 4893 9	FP	14	BTA14_r egion1	ARS-BFGL-NGS-4 939	4.29E-86	8.89E-87	2.18E-86	Yes	Yes	Core Candidate Genes	This study is the first to identify this gene in the context of heat stress/THI interactions in dairy cattle.	Not reported (new candidate)
23	LY6 D	FP	14	BTA14_r egion1	ARS-BFGL-NGS-4 939	4.29E-86	8.89E-87	2.18E-86	Yes	Yes	Core Candidate Genes	This study is the first to identify this gene in the context of heat stress/THI interactions in dairy cattle.	Not reported (new candidate)
24	LOC 1019 0522 2	FP	14	BTA14_r egion1	ARS-BFGL-NGS-4 939	4.29E-86	8.89E-87	2.18E-86	Yes	Yes	Core Candidate Genes	This study is the first to identify this gene in the context of heat stress/THI interactions in dairy cattle.	Not reported (new candidate)
25	AD GRB 1	FP	14	BTA14_r egion1	ARS-BFGL-NGS-4 939	4.29E-86	8.89E-87	2.18E-86	Yes	Yes	Core Candidate Genes	Prakapenka et al., 2024	Reported
26	LOC 1019 0191 8	FP	14	BTA14_r egion1	ARS-BFGL-NGS-4 939	4.29E-86	8.89E-87	2.18E-86	Yes	Yes	Core Candidate Genes	This study is the first to identify this gene in the context of heat stress/THI interactions in dairy cattle.	Not reported (new candidate)
27	GPR 20	FP	14	BTA14_r egion1	ARS-BFGL-NGS-4 939	4.29E-86	8.89E-87	2.18E-86	Yes	Yes	Core Candidate Genes	This study is the first to identify this	Not reported (new candidate)

												gene in the context of heat stress/THI interactions in dairy cattle.	
28	PTK2	FP	14	BTA14_region1	ARS-BFGL-NGS-4939	4.29E-86	8.89E-87	2.18E-86	Yes	Yes	Core Candidate Genes	This study is the first to identify this gene in the context of heat stress/THI interactions in dairy cattle.	Not reported (new candidate)
29	AGO2	FP	14	BTA14_region1	ARS-BFGL-NGS-4939	4.29E-86	8.89E-87	2.18E-86	Yes	Yes	Core Candidate Genes	This study is the first to identify this gene in the context of heat stress/THI interactions in dairy cattle.	Not reported (new candidate)
30	TRAPPC9	FP	14	BTA14_region1	UFL-rs134432442	1.26E-16	3.81E-17	7.03E-17	Yes	Yes	Core Candidate Genes	Teng et al., 2025	Reported
31	FAM135B	FP	14	BTA14_region1	ARS-BFGL-NGS-4939	4.29E-86	8.89E-87	2.18E-86	Yes	Yes	Core Candidate Genes	This study is the first to identify this gene in the context of heat stress/THI interactions in dairy cattle.	Not reported (new candidate)
32	LOC787350	FP	14	BTA14_region1	BovineHD1400024034	2.02E-08	2.22E-08	6.47E-09	Yes	Yes	Core Candidate Genes	This study is the first to propose a link between this and its interaction with heat stress/THI	Sparse reports
33	LOC112449570	FP	14	BTA14_region2	BovineHD1400001789	3.32E-06	NS	NS	No	No	Suggestive candidate genes	This study is the first to identify this gene in the context of heat	Not reported (new candidate)

												stress/THI interactions in dairy cattle.	
34	MGST1	FP	5	BTA5_region2	BovineHD0500026662	6.57E-11	5.64E-11	3.34E-11	Yes	Yes	Core candidate genes	Littlejohn et al., 2016; Raven et al., 2014; Fang et al., 2014; Iung et al., 2019	Reported
35	EPS8	FP	5	BTA5_region2	BovineHD0500026662	6.57E-11	5.64E-11	3.34E-11	Yes	Yes	Core Candidate Genes	Raven et al., 2014; Bohlouli et al., 2022	Reported
36	SLC15A5	FP	5	BTA5_region2	BovineHD0500026662	6.57E-11	5.64E-11	3.34E-11	Yes	Yes	Core Candidate Genes	Raven et al., 2014; Fang et al., 2014	Reported
37	LOC101903754	FP	16	BTA16_region1	ARS-BFGL-NGS-109702	1.08E-06	3.15E-09	1.01E-06	Yes	Yes	Core Candidate Genes	This study is the first to propose a link between this and its interaction with heat stress/THI	Sparse reports
38	KCNC2	PP	5	BTA5_region1	Hapmap44984-BTA-72893	1.29E-06	2.41E-06	2.02E-06	Yes	Yes	Core Candidate Genes	This study is the first to propose a link between this and its interaction with heat stress/THI	Sparse reports
39	GRID2	PP	6	BTA6_region1	Hapmap42776-BTA-105028	7.54E-07	3.08E-07	6.11E-07	Yes	Yes	Core Candidate Genes	This study is the first to propose a link between this and its interaction with heat stress/THI	Sparse reports
40	HERC3	PP	6	BTA6_region2	BovineHD4100004508	2.06E-15	2.31E-15	8.46E-16	Yes	Yes	Core Candidate Genes	This study is the first to identify this gene in the context of heat stress/THI interactions in dairy cattle.	Not reported (new candidate)
41	PIG	PP	6	BTA6_re	BovineHD4100004	2.06E-15	2.31E-15	8.46E-16	Yes	Yes	Core	This study is	Not reported (new

	Y			gion2	508						Candidate Genes	the first to identify this gene in the context of heat stress/THI interactions in dairy cattle.	candidate)
42	PYURF	PP	6	BTA6_region2	BovineHD4100004508	2.06E-15	2.31E-15	8.46E-16	Yes	Yes	Core Candidate Genes	This study is the first to identify this gene in the context of heat stress/THI interactions in dairy cattle.	Not reported (new candidate)
43	HERC6	PP	6	BTA6_region2	BovineHD4100004508	2.06E-15	2.31E-15	8.46E-16	Yes	Yes	Core Candidate Genes	This study is the first to identify this gene in the context of heat stress/THI interactions in dairy cattle.	Not reported (new candidate)
44	ABCG2	PP	6	BTA6_region2	BovineHD4100004508	2.06E-15	2.31E-15	8.46E-16	Yes	Yes	Core Candidate Genes	Cohen-Zinder et al., 2005; Iung et al., 2019	Reported
45	PKD2	PP	6	BTA6_region2	BovineHD0600010569	7.33E-19	1.09E-18	3.22E-19	Yes	Yes	Core Candidate Genes	Cohen-Zinder et al., 2005; Iung et al., 2019	Reported
46	LOC112447053	PP	6	BTA6_region2	BovineHD4100004508	2.06E-15	2.31E-15	8.46E-16	Yes	Yes	Core Candidate Genes	This study is the first to identify this gene in the context of heat stress/THI interactions in dairy cattle.	Not reported (new candidate)
47	IBSP	PP	6	BTA6_region2	BovineHD4100004508	2.06E-15	2.31E-15	8.46E-16	Yes	Yes	Core Candidate Genes	This study is the first to identify this gene in the context of heat stress/THI interactions in	Not reported (new candidate)

												dairy cattle.	
48	KCNS2	PP	14	BTA14_region3	BovineHD1400018987	3.13E-06	NS	2.46E-06	No	Yes	Potential Candidate Genes	This study is the first to identify this gene in the context of heat stress/THI interactions in dairy cattle.	Not reported (new candidate)
49	FRMD4B	PP	20	BTA20_region1	UA-IFASA-7069	1.38E-17	7.08E-18	3.47E-15	Yes	Yes	Core Candidate Genes	Teng et al., 2025	Reported
50	FAM19A1	PP	20	BTA20_region1	ARS-BFGL-NGS-26909	4.17E-10	2.06E-10	2.17E-08	Yes	Yes	Core Candidate Genes	Teng et al., 2025	Reported
51	WDR70	PP	20	BTA20_region2	BovineHD2000010588	1.22E-07	1.84E-07	1.24E-06	Yes	Yes	Core Candidate Genes	Teng et al., 2025	Reported
52	ADAMTS12	PP	20	BTA20_region3	BovineHD2000011414	1.29E-06	8.63E-07	3.02E-06	Yes	Yes	Core Candidate Genes	Teng et al., 2025	Reported

1. Candidate gene annotations are based on the bovine reference genome ARS-UCD1.2 and the Ensembl database (release 109, downloaded in April 2023); all genes within a 500-kb window upstream and downstream of each significant SNP were included in the candidate gene list.
2. "T support" and "RH support" indicate whether the SNP reached nominal significance ($P < 0.05$) in the temperature-interaction GWAS and humidity-interaction GWAS, respectively.
3. Core candidate genes: significant in the THI-GWAS and reaching nominal significance in both temperature and humidity GWAS (50 in total, including DGAT1, CPSF1, ABCG2, KCNC2, etc.).
Potential candidate genes: significant in the THI-GWAS and reaching nominal significance in either temperature or humidity GWAS, but not both (1 in total: KCNS2 on BTA14).
Suggestive candidate genes: significant in the THI-GWAS but failing to reach nominal significance in either temperature or humidity GWAS (1 in total: LOC112449570).
4. "NS" indicates that the gene did not reach nominal significance ($P \geq 0.05$). A dash (“--”) indicates that there is no clear representative SNP in the region where the gene is located.
5. This classification is based on statistical evidence and does not represent a ranking of functional importance; the functions of all candidate genes require validation through subsequent molecular biology experiments.
6. Genes associated with multiple traits (CPSF1, DGAT1) are listed in the "Trait" column, separated by commas.
7. The "Reports on Heat Stress/THI Interactions" column is divided into three categories:
Reported: clearly documented in heat stress or THI interaction studies in dairy cattle;
Sparse reports: very limited reports in dairy cattle heat stress or THI interaction studies, or reported in milk production trait GWAS but with heat stress/THI association first proposed in this study (6 in total: ZNF250, GRID2, LOC112449570, LOC112449590, LOC101903754, LOC787350). Note that KCNC2 on BTA5 was also rarely reported but is classified as a core candidate gene based on its statistical evidence across all three environmental dimensions (THI, temperature, and humidity).

Table S7. Genome-wide significant SNP×temperature interaction loci identified by temperature-interaction GWAS

Trait	Chr	SNP_ID	Physical location (bp)	P_value	MAF	Nearest genes	Does it overlap with the THI-GWAS
MY	14	BovineHD1400000188	403312	7.43E-10	0.2371	CPSF1	Yes
MY	14	ARS-BFGL-NGS-57820	465742	1.43E-09	0.236238	CPSF1	Yes
MY	14	BovineHD1400000206	494621	1.82E-10	0.215446	CPSF1	Yes
MY	14	UFL-rs134432442	550784	1.22E-10	0.214253	CPSF1	Yes
MY	14	ARS-BFGL-NGS-4939	609870	1.16E-10	0.215015	DGAT1	Yes
MY	1	ARS-BFGL-NGS-306	49040933	1.78E-07	0.0136955		No
FP	3	BovineHD0300021149	71884330	2.98E-07	0.124784		No
FP	5	BovineHD0500026533	93061021	1.30E-06	0.193063	MGST1	Yes
FP	5	BovineHD0500026557	93124633	8.03E-08	0.485144	MGST1	Yes
FP	5	BovineHD0500026578	93221107	1.24E-06	0.351638	MGST1	No
FP	5	BovineHD0500026623	93373699	3.60E-06	0.299178	MGST1	No
FP	5	BovineHD0500026635	93413676	2.69E-08	0.128565	MGST1	Yes
FP	5	BovineHD0500026655	93503991	7.64E-08	0.423067	MGST1	Yes
FP	5	BovineHD0500026662	93515983	5.64E-11	0.316985	MGST1	Yes
FP	5	BovineHD0500026668	93524134	6.30E-10	0.480236	MGST1	Yes
FP	5	BovineHD0500026682	93566261	5.27E-09	0.429036	MGST1	Yes
FP	5	BovineHD0500026721	93679644	1.23E-07	0.494363	MGST1	Yes
FP	5	Hapmap41349-BTA-74576	94169217	1.04E-06	0.0810121		Yes
FP	7	ARS-BFGL-NGS-32106	7513333	3.64E-06	0.45646		No
FP	7	BovineHD0700024290	80647410	2.55E-06	0.224466		No
FP	7	BTB-01483377	80686312	4.05E-06	0.273511		No
FP	14	BovineHD1400024339	146715	3.94E-17	0.468464	DGAT1	Yes
FP	14	BovineHD1400000138	223706	7.30E-15	0.484481	DGAT1	Yes
FP	14	BovineHD1400000143	243959	2.59E-13	0.479407	DGAT1	Yes
FP	14	BovineHD1400000152	255765	3.41E-10	0.447738	DGAT1	Yes
FP	14	Hapmap30381-BTC-005750	279949	2.27E-12	0.298945	DGAT1	Yes
FP	14	Hapmap30383-BTC-005848	305780	2.51E-27	0.353694	DGAT1	Yes
FP	14	BTA-34956-no-rs	330256	6.52E-11	0.474035	DGAT1	Yes
FP	14	BovineHD1400000187	399818	1.35E-08	0.463656	DGAT1	Yes
FP	14	BovineHD1400000188	403312	1.57E-61	0.2371	DGAT1/CPSF1	Yes
FP	14	ARS-BFGL-NGS-57820	465742	4.65E-60	0.236238	DGAT1/CPSF1	Yes
FP	14	ARS-BFGL-NGS-34135	490055	8.68E-25	0.384832	DGAT1/CPSF1	Yes
FP	14	BovineHD1400000206	494621	3.19E-69	0.215446	DGAT1/CPSF1	Yes

FP	14	ARS-BFGL-NGS-94706	511247	1.01E-25	0.397831	DGAT1/CPSF1	Yes
FP	14	UFL-rs134432442	550784	2.57E-85	0.214253	DGAT1/CPSF1	Yes
FP	14	ARS-BFGL-NGS-4939	609870	8.89E-87	0.215015	DGAT1	Yes
FP	14	BovineHD1400000243	676575	1.47E-29	0.39478	DGAT1	Yes
FP	14	BovineHD1400000246	688317	1.13E-28	0.399655	DGAT1	Yes
FP	14	BovineHD1400000249	700497	1.05E-28	0.399622	DGAT1	Yes
FP	14	Hapmap52798-ss46526455	731230	1.76E-18	0.442864	DGAT1	Yes
FP	14	ARS-BFGL-NGS-71749	762253	2.47E-08	0.185137		Yes
FP	14	BovineHD1400000262	775260	8.66E-50	0.247679	DGAT1	Yes
FP	14	UA-IFASA-6878	810863	7.61E-19	0.413782	DGAT1	Yes
FP	14	ARS-BFGL-NGS-107379	862483	1.59E-53	0.263099	DGAT1	Yes
FP	14	BovineHD1400000286	876529	1.11E-52	0.256864	DGAT1	Yes
FP	14	BovineHD1400000288	891340	6.62E-15	0.366196	DGAT1	Yes
FP	14	ARS-BFGL-NGS-18365	924727	8.30E-12	0.319505	DGAT1	Yes
FP	14	Hapmap30922-BTC-002021	946190	2.17E-09	0.293772	DGAT1	Yes
FP	14	BovineHD1400000301	959635	2.47E-08	0.353164	DGAT1	Yes
FP	14	BovineHD1400000305	973229	8.07E-08	0.331576	DGAT1	Yes
FP	14	Hapmap25384-BTC-001997	1022847	2.97E-12	0.466342	DGAT1	Yes
FP	14	Hapmap24715-BTC-001973	1044777	4.31E-13	0.460771	DGAT1	Yes
FP	14	BovineHD1400000325	1070218	1.53E-06	0.104689	DGAT1	No
FP	14	BTA-35941-no-rs	1082140	7.92E-15	0.43464	DGAT1	Yes
FP	14	ARS-BFGL-NGS-101653	1119139	1.80E-08	0.21611	DGAT1	Yes
FP	14	BovineHD1400000341	1142313	4.54E-16	0.428571	DGAT1	Yes
FP	14	ARS-BFGL-NGS-26520	1184910	4.74E-13	0.30508	DGAT1	Yes
FP	14	BovineHD4100010534	1197094	3.48E-16	0.429102	DGAT1	Yes
FP	14	Hapmap30086-BTC-002066	1322326	2.06E-19	0.473538	DGAT1	Yes
FP	14	BovineHD1400000397	1344741	1.01E-14	0.441935	DGAT1	Yes
FP	14	Hapmap30646-BTC-002054	1351419	3.23E-13	0.394084	DGAT1	Yes
FP	14	Hapmap25486-BTC-072553	1567838	4.86E-08	0.258854	DGAT1	Yes
FP	14	BovineHD1400000447	1610072	4.14E-09	0.485774	DGAT1	Yes
FP	14	ARS-BFGL-NGS-103064	1620025	2.70E-09	0.485708	DGAT1	Yes
FP	14	BovineHD1400000453	1657677	4.20E-10	0.352766	DGAT1	Yes
FP	14	ARS-BFGL-NGS-22866	1681783	3.65E-08	0.368683	DGAT1	Yes
FP	14	BovineHD1400000467	1747091	3.01E-19	0.42751	DGAT1	Yes
FP	14	ARS-BFGL-NGS-18858	1758505	6.30E-20	0.426084	DGAT1	Yes
FP	14	BovineHD1400000480	1785051	1.87E-08	0.4743	DGAT1	Yes

FP	14	ARS-BFGL-NGS-59769	1854457	1.39E-08	0.462694	DGAT1	Yes
FP	14	BovineHD1400024350	1896438	2.48E-10	0.350146	DGAT1	Yes
FP	14	Hapmap36620-SCAFFOLD50018_7571	2142001	4.67E-13	0.461169	DGAT1	Yes
FP	14	BovineHD1400000616	2216794	3.80E-24	0.482657	DGAT1	Yes
FP	14	BovineHD1400000654	2309238	1.30E-07	0.28074	DGAT1	Yes
FP	14	ARS-BFGL-NGS-74378	2616322	3.93E-09	0.325872	DGAT1	Yes
FP	14	ARS-BFGL-NGS-117542	2640274	9.63E-07	0.232226	DGAT1	No
FP	14	BovineHD1400000755	2719625	8.67E-07	0.279977	DGAT1	No
FP	14	BovineHD1400000779	2805476	2.44E-06	0.299144	DGAT1	No
FP	14	BovineHD1400000788	2840340	8.17E-09	0.446313	DGAT1	Yes
FP	14	BovineHD1400000792	2867641	8.48E-08	0.383406	DGAT1	Yes
FP	14	UA-IFASA-9288	2929132	3.69E-14	0.307667	DGAT1	Yes
FP	14	Hapmap32970-BTC-064990	3015800	1.74E-07	0.3847	DGAT1	Yes
FP	14	Hapmap24986-BTC-065021	3040885	2.83E-07	0.384003	DGAT1	Yes
FP	14	BovineHD1400000870	3106715	6.74E-09	0.492605	DGAT1	Yes
FP	14	Hapmap26072-BTC-065132	3120000	1.83E-07	0.307866	DGAT1	Yes
FP	14	Hapmap26527-BTC-005059	3147254	1.03E-07	0.311878	DGAT1	Yes
FP	14	ARS-BFGL-NGS-56327	3308936	6.14E-08	0.38533	DGAT1	Yes
FP	14	ARS-BFGL-NGS-100480	3336128	2.28E-14	0.453707	DGAT1	Yes
FP	14	BovineHD1400000977	3385942	4.84E-07	0.378664	DGAT1	Yes
FP	14	BovineHD1400000999	3427682	1.89E-06	0.410996	DGAT1	Yes
FP	14	UA-IFASA-5306	3439565	4.06E-15	0.320003	DGAT1	Yes
FP	14	Hapmap27703-BTC-053907	3554540	4.83E-08	0.350511	DGAT1	Yes
FP	14	BovineHD4100010659	3730901	8.16E-07	0.303489	DGAT1	Yes
FP	14	Hapmap23302-BTC-052123	3819126	2.67E-06	0.29364	DGAT1	Yes
FP	14	ARS-BFGL-NGS-3571	4188954	4.73E-07	0.438918	DGAT1	Yes
FP	14	BovineHD4100010689	4257911	1.07E-06	0.495656	DGAT1	Yes
FP	14	ARS-BFGL-NGS-115947	4459231	1.38E-07	0.420082	DGAT1	Yes
FP	16	ARS-BFGL-NGS-109702	8086136	3.15E-09	0.044303	LOC101903754	Yes
FP	14	BovineHD1400024034	52575	2.22E-08	0.18328	LOC787350	Yes
PP	5	Hapmap44984-BTA-72893	4517412	2.41E-06	0.365533	KCNC2	No
PP	6	Hapmap42776-BTA-105028	31067757	3.08E-07	0.0108105	GRID2	Yes
PP	6	BovineHD4100004508	36186105	2.31E-15	0.0285515	PKD2	Yes
PP	6	Hapmap24324-BTC-062449	36205216	2.10E-15	0.0286842	PKD2	Yes

PP	6	BovineHD4100004514	36218002	1.74E-15	0.0283857	PKD2	Yes
PP	6	BovineHD4100004517	36241100	1.68E-12	0.0343878	PKD2	Yes
PP	6	BovineHD0600010428	36251003	4.15E-15	0.0285184	PKD2	Yes
PP	6	BovineHD4100004526	36314103	5.73E-14	0.0295795	PKD2	Yes
PP	6	BovineHD0600010444	36326417	1.36E-15	0.0294469	PKD2	Yes
PP	6	BovineHD0600010445	36330445	2.27E-07	0.0320666	PKD2	Yes
PP	6	BovineHD4100004528	36332467	1.79E-13	0.031337	PKD2	Yes
PP	6	BovineHD4100004529	36334803	1.93E-15	0.0287173	PKD2	Yes
PP	6	BovineHD4100004530	36342054	9.72E-16	0.0287837	PKD2	Yes
PP	6	BovineHD4100004531	36364608	1.52E-15	0.0303091	PKD2	Yes
PP	6	BovineHD0600010555	36592235	5.84E-11	0.087379	PKD2	Yes
PP	6	BTA-121739-no-rs	36635943	7.82E-11	0.113045	PKD2	Yes
PP	6	BovineHD0600010569	36657367	1.09E-18	0.0478512	PKD2	Yes
PP	6	BovineHD0600010605	36855289	1.35E-08	0.232557	PKD2	Yes
PP	6	Hapmap26259-BTC-033526	36892307	1.91E-07	0.261706	PKD2	Yes
PP	6	BovineHD4100004553	36915121	1.06E-06	0.289064	PKD2	Yes
PP	6	BovineHD0600010624	36942473	3.90E-16	0.11414	PKD2	Yes
PP	6	BovineHD0600010625	36943641	2.64E-10	0.179599	PKD2	Yes
PP	6	BovineHD4100004557	36944790	2.10E-10	0.181755	PKD2	Yes
PP	6	BovineHD4100004558	36956973	5.36E-15	0.0576336	PKD2	Yes
PP	6	BovineHD0600010630	36970336	4.88E-17	0.0538533	PKD2	Yes
PP	6	BovineHD4100004560	36972388	9.66E-17	0.0550471	PKD2	Yes
PP	6	BovineHD0600010650	37042156	8.04E-07	0.453343		No
PP	14	BovineHD1400000138	223706	1.56E-06	0.484481	DGAT1	Yes
PP	14	BovineHD1400000188	403312	1.80E-12	0.2371	DGAT1/CPSF1	Yes
PP	14	ARS-BFGL-NGS-57820	465742	1.27E-11	0.236238	DGAT1/CPSF1	Yes
PP	14	ARS-BFGL-NGS-34135	490055	2.51E-07	0.384832	DGAT1/CPSF1	Yes
PP	14	BovineHD1400000206	494621	1.29E-14	0.215446	DGAT1/CPSF1	Yes
PP	14	ARS-BFGL-NGS-94706	511247	1.13E-07	0.397831	DGAT1/CPSF1	Yes
PP	14	UFL-rs134432442	550784	3.81E-17	0.214253	DGAT1/CPSF1	Yes
PP	14	ARS-BFGL-NGS-4939	609870	8.56E-17	0.215015	DGAT1	Yes
PP	14	BovineHD1400000262	775260	1.01E-09	0.247679	DGAT1	Yes
PP	14	ARS-BFGL-NGS-107379	862483	4.71E-10	0.263099	DGAT1	Yes
PP	14	BovineHD1400000286	876529	2.53E-10	0.256864	DGAT1	Yes
PP	14	Hapmap30086-BTC-002066	1322326	2.07E-06	0.473538	DGAT1	Yes
PP	20	BovineHD2000009025	31228123	1.04E-07	0.222112	FRMD4B	Yes

PP	20	BovineHD2000009068	31423773	2.47E-07	0.425388	FRMD4B	No
PP	20	BovineHD2000009105	31591442	3.38E-08	0.173498	FRMD4B	Yes
PP	20	BovineHD2000009188	31890580	9.14E-11	0.492539	FRMD4B	Yes
PP	20	UA-IFASA-7069	31912365	7.08E-18	0.343779	FRMD4B	Yes
PP	20	BovineHD2000009204	31963910	6.70E-11	0.494495	FRMD4B	Yes
PP	20	BovineHD2000009215	31980361	1.05E-10	0.494429	FRMD4B	Yes
PP	20	ARS-BFGL-NGS-118998	32009781	1.79E-16	0.351307	FRMD4B	Yes
PP	20	BovineHD2000009226	32025240	1.88E-17	0.348819	FRMD4B	Yes
PP	20	BovineHD2000009234	32035193	2.64E-17	0.348422	FRMD4B	Yes
PP	20	BovineHD2000009251	32077537	3.25E-16	0.350511	FRMD4B	Yes
PP	20	BovineHD2000009262	32127333	3.59E-09	0.480568	FRMD4B	Yes
PP	20	BovineHD2000009307	32372127	2.91E-08	0.293209	FRMD4B	Yes
PP	20	BovineHD2000009318	32437127	3.46E-06	0.410432		No
PP	20	BovineHD2000009361	32694119	1.56E-07	0.414478	FAM19A1	Yes
PP	20	BovineHD2000009459	33024411	2.16E-11	0.366428	FAM19A1	Yes
PP	20	BovineHD2000009565	33341878	2.70E-06	0.138049		No
PP	20	ARS-BFGL-NGS-26909	33537716	2.06E-10	0.218729	FAM19A1	Yes
PP	20	BovineHD2000009668	33541612	2.42E-06	0.273047	FAM19A1	Yes
PP	20	BovineHD2000009742	33809571	3.49E-07	0.138977	FAM19A1	Yes
PP	20	BovineHD2000010588	36900514	1.84E-07	0.161726	WDR70	Yes
PP	20	BovineHD2000011414	39896176	8.63E-07	0.144117	ADAMTS12	Yes
PP	20	BTB-00785931	45766550	3.13E-06	0.180561		No

Table S8. Genome-wide significant SNP×relative humidity interaction loci identified by relative humidity-interaction GWAS

Trait	Chr	SNP_ID	Physical location(bp)	P_value	MAF	Nearest genes	Does it overlap with the THI-GWAS
MY	14	BovineHD1400000188	403312	2.40E-10	0.2371	CPSF1	Yes
MY	14	ARS-BFGL-NGS-57820	465742	4.85E-10	0.236238	CPSF1	Yes
MY	14	BovineHD1400000206	494621	5.46E-11	0.215446	CPSF1	Yes
MY	14	UFL-rs134432442	550784	6.60E-11	0.214253	CPSF1	Yes
MY	14	ARS-BFGL-NGS-4939	609870	4.90E-11	0.215015	DGAT1	Yes
MY	1	ARS-BFGL-NGS-306	49040933	2.82E-07	0.0136955		No
FP	5	BovineHD0500026533	93061021	2.39E-06	0.193063	MGST1	Yes
FP	5	BovineHD0500026557	93124633	3.48E-08	0.485144	MGST1	Yes
FP	5	BovineHD0500026578	93221107	3.36E-06	0.351638	MGST1	No
FP	5	BovineHD0500026623	93373699	1.76E-06	0.299178	MGST1	No
FP	5	BovineHD0500026635	93413676	1.12E-07	0.128565	MGST1	Yes
FP	5	BovineHD0500026655	93503991	2.07E-08	0.423067	MGST1	Yes

FP	5	BovineHD0500026662	93515983	3.34E-11	0.316985	MGST1	Yes
FP	5	BovineHD0500026668	93524134	2.24E-10	0.480236	MGST1	Yes
FP	5	BovineHD0500026682	93566261	9.48E-09	0.429036	MGST1	Yes
FP	5	BovineHD0500026721	93679644	3.60E-08	0.494363	MGST1	Yes
FP	5	Hapmap41349-BTA-74576	94169217	3.03E-06	0.0810121		Yes
FP	5	BovineHD0500026872	94193739	8.16E-08	0.13911		No
FP	5	Hapmap53294-rs29016908	94217230	5.17E-09	0.219691		No
FP	14	BovineHD1400024339	146715	3.98E-17	0.468464	DGAT1	Yes
FP	14	BovineHD1400000138	223706	1.94E-15	0.484481	DGAT1	Yes
FP	14	BovineHD1400000143	243959	4.80E-14	0.479407	DGAT1	Yes
FP	14	BovineHD1400000152	255765	2.76E-10	0.447738	DGAT1	Yes
FP	14	Hapmap30381-BTC-005750	279949	5.98E-13	0.298945	DGAT1	Yes
FP	14	Hapmap30383-BTC-005848	305780	1.08E-27	0.353694	DGAT1	Yes
FP	14	BTA-34956-no-rs	330256	1.05E-11	0.474035	DGAT1	Yes
FP	14	BovineHD1400000187	399818	1.25E-09	0.463656	DGAT1	Yes
FP	14	BovineHD1400000188	403312	3.42E-61	0.2371	DGAT1/CPSF1	Yes
FP	14	ARS-BFGL-NGS-57820	465742	3.21E-60	0.236238	DGAT1/CPSF1	Yes
FP	14	ARS-BFGL-NGS-34135	490055	3.24E-25	0.384832	DGAT1/CPSF1	Yes
FP	14	BovineHD1400000206	494621	1.66E-69	0.215446	DGAT1/CPSF1	Yes
FP	14	ARS-BFGL-NGS-94706	511247	1.11E-25	0.397831	DGAT1/CPSF1	Yes
FP	14	UFL-rs134432442	550784	4.97E-85	0.214253	DGAT1/CPSF1	Yes
FP	14	ARS-BFGL-NGS-4939	609870	2.18E-86	0.215015	DGAT1	Yes
FP	14	BovineHD1400000243	676575	1.64E-29	0.39478	DGAT1	Yes
FP	14	BovineHD1400000246	688317	2.17E-28	0.399655	DGAT1	Yes
FP	14	BovineHD1400000249	700497	2.30E-28	0.399622	DGAT1	Yes
FP	14	Hapmap52798-ss46526455	731230	6.43E-19	0.442864	DGAT1	Yes
FP	14	ARS-BFGL-NGS-71749	762253	1.25E-08	0.185137		Yes
FP	14	BovineHD1400000262	775260	5.23E-50	0.247679	DGAT1	Yes
FP	14	UA-IFASA-6878	810863	6.30E-20	0.413782	DGAT1	Yes
FP	14	ARS-BFGL-NGS-107379	862483	1.08E-53	0.263099	DGAT1	Yes
FP	14	BovineHD1400000286	876529	6.37E-53	0.256864	DGAT1	Yes
FP	14	BovineHD1400000288	891340	1.91E-15	0.366196	DGAT1	Yes
FP	14	ARS-BFGL-NGS-18365	924727	4.81E-12	0.319505	DGAT1	Yes
FP	14	Hapmap30922-BTC-002021	946190	4.31E-10	0.293772	DGAT1	Yes
FP	14	BovineHD1400000301	959635	5.17E-09	0.353164	DGAT1	Yes

FP	14	BovineHD1400000305	973229	1.05E-08	0.331576	DGAT1	Yes
FP	14	Hapmap25384-BTC-001997	1022847	8.28E-12	0.466342	DGAT1	Yes
FP	14	Hapmap24715-BTC-001973	1044777	3.02E-12	0.460771	DGAT1	Yes
FP	14	BovineHD1400000325	1070218	2.77E-06	0.104689	DGAT1	Yes
FP	14	BTA-35941-no-rs	1082140	2.03E-15	0.43464	DGAT1	Yes
FP	14	ARS-BFGL-NGS-101653	1119139	4.82E-09	0.21611	DGAT1	Yes
FP	14	BovineHD1400000341	1142313	1.36E-16	0.428571	DGAT1	Yes
FP	14	ARS-BFGL-NGS-26520	1184910	3.20E-13	0.30508	DGAT1	Yes
FP	14	BovineHD4100010534	1197094	1.31E-16	0.429102	DGAT1	Yes
FP	14	Hapmap30086-BTC-002066	1322326	3.48E-19	0.473538	DGAT1	Yes
FP	14	BovineHD1400000397	1344741	7.38E-15	0.441935	DGAT1	Yes
FP	14	Hapmap30646-BTC-002054	1351419	1.91E-13	0.394084	DGAT1	Yes
FP	14	Hapmap25486-BTC-072553	1567838	4.62E-08	0.258854	DGAT1	Yes
FP	14	BovineHD1400000447	1610072	1.00E-08	0.485774	DGAT1	Yes
FP	14	ARS-BFGL-NGS-103064	1620025	6.17E-09	0.485708	DGAT1	Yes
FP	14	BovineHD1400000453	1657677	2.04E-10	0.352766	DGAT1	Yes
FP	14	ARS-BFGL-NGS-22866	1681783	1.75E-08	0.368683	DGAT1	Yes
FP	14	BovineHD1400000467	1747091	9.33E-20	0.42751	DGAT1	Yes
FP	14	ARS-BFGL-NGS-18858	1758505	2.44E-20	0.426084	DGAT1	Yes
FP	14	BovineHD1400000480	1785051	7.92E-09	0.4743	DGAT1	Yes
FP	14	ARS-BFGL-NGS-59769	1854457	4.89E-08	0.462694	DGAT1	Yes
FP	14	BovineHD1400024350	1896438	2.52E-10	0.350146	DGAT1	Yes
FP	14	Hapmap36620-SCAFFOLD50018_7571	2142001	4.16E-13	0.461169	DGAT1	Yes
FP	14	BovineHD1400000616	2216794	2.02E-23	0.482657	DGAT1	Yes
FP	14	BovineHD1400000654	2309238	1.34E-08	0.28074	DGAT1	Yes
FP	14	ARS-BFGL-NGS-74378	2616322	4.62E-09	0.325872	DGAT1	Yes
FP	14	ARS-BFGL-NGS-117542	2640274	1.42E-08	0.232226	DGAT1	No
FP	14	BovineHD1400000755	2719625	3.21E-07	0.279977	DGAT1	No
FP	14	BovineHD1400000779	2805476	2.44E-06	0.299144	DGAT1	No
FP	14	BovineHD1400000788	2840340	1.43E-09	0.446313	DGAT1	Yes
FP	14	BovineHD1400000792	2867641	1.04E-07	0.383406	DGAT1	Yes
FP	14	UA-IFASA-9288	2929132	1.14E-13	0.307667	DGAT1	Yes
FP	14	Hapmap32970-BTC-064990	3015800	4.94E-07	0.3847	DGAT1	Yes
FP	14	Hapmap24986-BTC-065021	3040885	7.96E-07	0.384003	DGAT1	Yes
FP	14	BovineHD1400000870	3106715	1.26E-08	0.492605	DGAT1	Yes
FP	14	Hapmap26072-BTC-06513	3120000	1.85E-08	0.307866	DGAT1	Yes

		2					
FP	14	Hapmap26527-BTC-005059	3147254	4.48E-09	0.311878	DGAT1	Yes
FP	14	ARS-BFGL-NGS-56327	3308936	4.97E-08	0.38533	DGAT1	Yes
FP	14	ARS-BFGL-NGS-100480	3336128	2.93E-14	0.453707	DGAT1	Yes
FP	14	BovineHD1400000977	3385942	1.08E-07	0.378664	DGAT1	Yes
FP	14	BovineHD1400000999	3427682	5.24E-07	0.410996	DGAT1	Yes
FP	14	UA-IFASA-5306	3439565	2.17E-15	0.320003	DGAT1	Yes
FP	14	Hapmap27703-BTC-053907	3554540	1.54E-08	0.350511	DGAT1	Yes
FP	14	BovineHD1400001053	3568280	1.83E-06	0.462694	DGAT1	No
FP	14	BovineHD4100010659	3730901	1.21E-06	0.303489	DGAT1	Yes
FP	14	ARS-BFGL-NGS-3571	4188954	8.17E-07	0.438918	DGAT1	Yes
FP	14	BovineHD4100010689	4257911	1.81E-06	0.495656	DGAT1	Yes
FP	14	ARS-BFGL-NGS-115947	4459231	1.64E-06	0.420082	DGAT1	Yes
FP	14	BovineHD1400024034	52575	6.47E-09	0.18328	LOC787350	Yes
FP	16	ARS-BFGL-NGS-109702	8086136	1.01E-06	0.044303	LOC101903754	Yes
PP	5	Hapmap44984-BTA-72893	4517412	2.02E-06	0.365533	KCNC2	Yes
PP	6	Hapmap42776-BTA-105028	31067757	6.11E-07	0.0108105	GRID2	Yes
PP	6	BovineHD4100004496	36132072	2.71E-07	0.250497	PKD2	No
PP	6	BovineHD4100004508	36186105	8.46E-16	0.0285515	PKD2	Yes
PP	6	Hapmap24324-BTC-062449	36205216	6.77E-16	0.0286842	PKD2	Yes
PP	6	BovineHD4100004514	36218002	6.49E-16	0.0283857	PKD2	Yes
PP	6	BovineHD4100004517	36241100	9.96E-13	0.0343878	PKD2	Yes
PP	6	BovineHD0600010428	36251003	1.49E-15	0.0285184	PKD2	Yes
PP	6	BovineHD0600010435	36296985	1.53E-06	0.121999	PKD2	No
PP	6	BovineHD4100004526	36314103	1.85E-14	0.0295795	PKD2	Yes
PP	6	BovineHD0600010444	36326417	6.23E-16	0.0294469	PKD2	Yes
PP	6	BovineHD0600010445	36330445	1.14E-07	0.0320666	PKD2	Yes
PP	6	BovineHD4100004528	36332467	6.78E-14	0.031337	PKD2	Yes
PP	6	BovineHD4100004529	36334803	9.02E-16	0.0287173	PKD2	Yes
PP	6	BovineHD4100004530	36342054	4.43E-16	0.0287837	PKD2	Yes
PP	6	BovineHD4100004531	36364608	8.40E-16	0.0303091	PKD2	Yes
PP	6	BovineHD0600010555	36592235	5.54E-09	0.087379	PKD2	Yes
PP	6	BTA-121739-no-rs	36635943	8.14E-13	0.113045	PKD2	Yes
PP	6	BovineHD0600010569	36657367	3.22E-19	0.0478512	PKD2	Yes
PP	6	BovineHD0600010605	36855289	6.75E-08	0.232557	PKD2	Yes
PP	6	Hapmap26259-BTC-033526	36892307	2.33E-06	0.261706	PKD2	Yes
PP	6	BovineHD0600010624	36942473	2.16E-15	0.11414	PKD2	Yes
PP	6	BovineHD0600010625	36943641	8.99E-12	0.179599	PKD2	Yes

PP	6	BovineHD4100004557	36944790	7.71E-12	0.181755	PKD2	Yes
PP	6	BovineHD4100004558	36956973	7.17E-14	0.0576336	PKD2	Yes
PP	6	BovineHD0600010630	36970336	1.18E-16	0.0538533	PKD2	Yes
PP	6	BovineHD4100004560	36972388	8.36E-16	0.0550471	PKD2	Yes
PP	7	BovineHD0700009741	32705861	5.30E-07	0.0372728		No
PP	11	BovineHD1100031136	105225539	2.32E-06	0.141498		No
PP	14	BovineHD1400000188	403312	1.71E-12	0.2371	DGAT1/CPSF1	Yes
PP	14	ARS-BFGL-NGS-57820	465742	6.10E-12	0.236238	DGAT1/CPSF1	Yes
PP	14	ARS-BFGL-NGS-34135	490055	1.15E-07	0.384832	DGAT1/CPSF1	Yes
PP	14	BovineHD1400000206	494621	4.83E-15	0.215446	DGAT1/CPSF1	Yes
PP	14	ARS-BFGL-NGS-94706	511247	4.20E-08	0.397831	DGAT1/CPSF1	Yes
PP	14	UFL-rs134432442	550784	7.03E-17	0.214253	DGAT1/CPSF1	Yes
PP	14	ARS-BFGL-NGS-4939	609870	1.05E-16	0.215015	DGAT1	Yes
PP	14	BovineHD1400000243	676575	2.66E-06	0.39478	DGAT1	Yes
PP	14	BovineHD1400000262	775260	3.01E-10	0.247679	DGAT1	Yes
PP	14	ARS-BFGL-NGS-107379	862483	2.92E-10	0.263099	DGAT1	Yes
PP	14	BovineHD1400000286	876529	1.48E-10	0.256864	DGAT1	Yes
PP	14	Hapmap30086-BTC-002066	1322326	2.66E-06	0.473538	DGAT1	Yes
PP	14	BovineHD1400018987	65795499	2.46E-06	0.0909603	KCNS2	Yes
PP	20	BovineHD2000009025	31228123	2.36E-07	0.222112	FRMD4B	Yes
PP	20	BovineHD2000009105	31591442	1.60E-07	0.173498	FRMD4B	Yes
PP	20	BovineHD2000009188	31890580	1.41E-09	0.492539	FRMD4B	Yes
PP	20	UA-IFASA-7069	31912365	3.47E-15	0.343779	FRMD4B	Yes
PP	20	BovineHD2000009204	31963910	9.67E-10	0.494495	FRMD4B	Yes
PP	20	BovineHD2000009215	31980361	1.45E-09	0.494429	FRMD4B	Yes
PP	20	ARS-BFGL-NGS-118998	32009781	8.59E-14	0.351307	FRMD4B	Yes
PP	20	BovineHD2000009226	32025240	1.99E-14	0.348819	FRMD4B	Yes
PP	20	BovineHD2000009234	32035193	4.76E-14	0.348422	FRMD4B	Yes
PP	20	BovineHD2000009251	32077537	1.62E-13	0.350511	FRMD4B	Yes
PP	20	BovineHD2000009262	32127333	1.08E-07	0.480568	FRMD4B	Yes
PP	20	BovineHD2000009307	32372127	3.13E-07	0.293209	FRMD4B	Yes
PP	20	BovineHD2000009361	32694119	1.90E-06	0.414478	FAM19A1	Yes
PP	20	BovineHD2000009459	33024411	5.30E-10	0.366428	FAM19A1	Yes
PP	20	ARS-BFGL-NGS-26909	33537716	2.17E-08	0.218729	FAM19A1	Yes
PP	20	BovineHD2000009742	33809571	2.35E-07	0.138977	FAM19A1	Yes
PP	20	BovineHD2000009909	34507773	3.00E-06	0.263463		No
PP	20	BovineHD2000010588	36900514	1.24E-06	0.161726	WDR70	Yes

PP	20	BovineHD2000011414	39896176	3.02E-06	0.144117	ADAMTS12	Yes
PP	22	ARS-BFGL-NGS-4455	52245623	2.03E-06	0.120706		No

Table S9. Genome-wide significant loci identified by conventional main-effect GWAS and their overlap with THI-interaction GWAS

Trait	Chr	SNP ID	Location(bp)	GWAS P-value for the main effect	THI-GWAS P-value	Overlap with THI-GWAS	Candidate genes
MY	1	BTB-00055938	122501524	9.22E-07		No	
FP	3	BTA-68670-no-rs	88521220	2.69E-06		No	
FP	5	BovineHD0500026400	92523198	1.50E-06		No	
FP	5	Hapmap34380-BES4_Contig293_1272	92527188	2.21E-06		No	
FP	5	BovineHD0500026557	93124633	2.34E-06	3.51E-08	Yes	MGST1
FP	5	BovineHD0500026635	93413676	2.39E-06	2.52E-08	Yes	MGST1
FP	5	BovineHD0500026662	93515983	5.94E-09	6.57E-11	Yes	MGST1
FP	5	BovineHD0500026668	93524134	1.98E-07	4.12E-10	Yes	MGST1
FP	5	BovineHD0500026721	93679644	3.85E-06	1.93E-07	Yes	MGST1
FP	14	BovineHD1400024339	146715	1.91E-08	2.56E-16	Yes	DGAT1
FP	14	BovineHD1400000138	223706	2.27E-08	1.02E-14	Yes	DGAT1
FP	14	BovineHD1400000143	243959	2.21E-08	5.54E-13	Yes	DGAT1
FP	14	Hapmap30381-BTC-005750	279949	5.02E-08	2.07E-12	Yes	DGAT1
FP	14	Hapmap30383-BTC-005848	305780	3.22E-17	1.99E-27	Yes	DGAT1
FP	14	BovineHD1400000188	403312	1.96E-41	1.11E-61	Yes	DGAT1, CPSF1
FP	14	ARS-BFGL-NGS-57820	465742	2.91E-39	3.53E-60	Yes	DGAT1, CPSF1
FP	14	ARS-BFGL-NGS-34135	490055	6.82E-13	1.76E-24	Yes	DGAT1, CPSF1
FP	14	BovineHD1400000206	494621	1.01E-47	5.66E-69	Yes	DGAT1, CPSF1
FP	14	ARS-BFGL-NGS-94706	511247	1.76E-12	2.20E-25	Yes	DGAT1, CPSF1
FP	14	UFL-rs134432442	550784	2.63E-67	1.18E-84	Yes	DGAT1, CPSF1
FP	14	ARS-BFGL-NGS-4939	609870	1.19E-68	4.29E-86	Yes	DGAT1
FP	14	BovineHD1400000243	676575	1.54E-24	1.02E-28	Yes	DGAT1
FP	14	BovineHD1400000246	688317	1.70E-23	1.00E-27	Yes	DGAT1
FP	14	BovineHD1400000249	700497	2.15E-23	9.50E-28	Yes	DGAT1
FP	14	Hapmap52798-ss46526455	731230	1.45E-12	4.23E-18	Yes	DGAT1

FP	14	ARS-BFGL-NGS-71749	762253	7.96E-07	1.86E-08	Yes	
FP	14	BovineHD1400000262	775260	1.95E-27	1.05E-49	Yes	DGAT1
FP	14	UA-IFASA-6878	810863	6.10E-13	1.21E-18	Yes	DGAT1
FP	14	ARS-BFGL-NGS-107379	862483	1.48E-38	3.96E-53	Yes	DGAT1
FP	14	BovineHD1400000286	876529	7.73E-38	2.65E-52	Yes	DGAT1
FP	14	BovineHD1400000288	891340	1.04E-09	9.32E-15	Yes	DGAT1
FP	14	ARS-BFGL-NGS-18365	924727	4.17E-07	1.76E-11	Yes	DGAT1
FP	14	BovineHD1400000325	1070218	2.14E-07	1.53E-06	Yes	DGAT1
FP	14	ARS-BFGL-NGS-101653	1119139	6.50E-07	1.63E-08	Yes	DGAT1
FP	14	ARS-BFGL-NGS-26520	1184910	6.13E-09	6.60E-13	Yes	DGAT1
FP	14	BovineHD4100010534	1197094	3.42E-06	3.57E-16	Yes	DGAT1
FP	14	Hapmap30086-BTC-002066	1322326	1.49E-08	3.29E-19	Yes	DGAT1
FP	14	BovineHD1400000397	1344741	3.14E-06	1.39E-14	Yes	DGAT1
FP	14	BovineHD1400000467	1747091	2.38E-08	5.53E-19	Yes	DGAT1
FP	14	ARS-BFGL-NGS-18858	1758505	8.40E-09	1.33E-19	Yes	DGAT1
FP	14	BovineHD1400000616	2216794	2.12E-11	2.12E-24	Yes	DGAT1
FP	14	Hapmap26527-BTC-005059	3147254	2.06E-07	1.22E-07	Yes	DGAT1
FP	14	UA-IFASA-5306	3439565	2.17E-06	2.49E-15	Yes	DGAT1
FP	20	UA-IFASA-7069	31912365	1.93E-06	1.38E-17	No	FRMD4B
FP	14	BovineHD1400024034	52575	1.69E-06	2.02E-08	Yes	LOC787350
PP	1	BTB-00055938	122501524	5.80E-13		No	
PP	4	ARS-BFGL-NGS-38960	117346685	3.45E-07		No	
PP	6	BovineHD4100004508	36186105	7.18E-12	2.06E-15	Yes	PKD2
PP	6	Hapmap24324-BTC-062449	36205216	2.13E-11	1.87E-15	Yes	PKD2
PP	6	BovineHD4100004514	36218002	1.61E-11	1.52E-15	Yes	PKD2
PP	6	BovineHD4100004517	36241100	1.61E-09	1.64E-12	Yes	PKD2
PP	6	BovineHD0600010428	36251003	1.54E-11	3.45E-15	Yes	PKD2
PP	6	BovineHD4100004526	36314103	1.66E-10	5.70E-14	Yes	PKD2
PP	6	BovineHD0600010444	36326417	2.68E-11	1.23E-15	Yes	PKD2
PP	6	BovineHD4100004528	36332467	2.65E-10	1.60E-13	Yes	PKD2
PP	6	BovineHD4100004529	36334803	3.37E-12	1.64E-15	Yes	PKD2
PP	6	BovineHD4100004530	36342054	1.65E-12	8.38E-16	Yes	PKD2
PP	6	BovineHD4100004531	36364608	1.28E-12	1.23E-15	Yes	PKD2
PP	6	BovineHD0600010555	36592235	4.44E-09	2.01E-10	Yes	PKD2
PP	6	BTA-121739-no-rs	36635943	6.20E-10	6.45E-11	Yes	PKD2
PP	6	BovineHD0600010569	36657367	6.27E-14	7.33E-19	Yes	PKD2
PP	6	BovineHD0600010605	36855289	1.53E-08	2.54E-08	Yes	PKD2
PP	6	Hapmap26259-BTC-033526	36892307	1.79E-06	3.15E-07	Yes	PKD2
PP	6	BovineHD4100004553	36915121	5.12E-07	2.46E-06	Yes	PKD2
PP	6	BovineHD0600010624	36942473	4.55E-13	5.47E-16	Yes	PKD2
PP	6	BovineHD0600010625	36943641	1.12E-10	1.98E-10	Yes	PKD2
PP	6	BovineHD4100004557	36944790	4.29E-11	1.48E-10	Yes	PKD2
PP	6	BovineHD4100004558	36956973	3.85E-11	4.16E-15	Yes	PKD2

PP	6	BovineHD0600010630	36970336	8.67E-14	3.31E-17	Yes	PKD2
PP	6	BovineHD4100004560	36972388	7.01E-12	7.01E-17	Yes	PKD2
PP	8	BovineHD0800028360	94306363	1.93E-09		No	
PP	9	BovineHD0900014881	53214330	2.80E-08		No	
PP	12	BovineHD1200009396	31919365	2.01E-17		No	
PP	14	BovineHD1400000188	403312	6.41E-10	5.72E-12	Yes	DGAT1, CPSF1
PP	14	ARS-BFGL-NGS-57820	465742	9.60E-10	3.06E-11	Yes	DGAT1, CPSF1
PP	14	BovineHD1400000206	494621	3.33E-10	4.13E-14	Yes	DGAT1, CPSF1
PP	14	ARS-BFGL-NGS-94706	511247	1.60E-06	3.57E-07	Yes	DGAT1, CPSF1
PP	14	UFL-rs134432442	550784	2.54E-11	1.26E-16	Yes	DGAT1, CPSF1
PP	14	ARS-BFGL-NGS-4939	609870	6.33E-11	3.03E-16	Yes	DGAT1
PP	14	BovineHD1400000262	775260	3.22E-06	2.10E-09	Yes	DGAT1
PP	14	ARS-BFGL-NGS-107379	862483	1.27E-08	2.26E-09	Yes	DGAT1
PP	14	BovineHD1400000286	876529	1.61E-07	1.18E-09	Yes	DGAT1
PP	14	BovineHD1400013782	46309357	3.90E-06		No	
PP	16	ARS-BFGL-NGS-109702	8086136	5.07E-08	1.08E-06	No	LOC10190 3754
PP	20	BovineHD2000009188	31890580	7.18E-08	1.98E-10	Yes	FRMD4B
PP	20	UA-IFASA-7069	31912365	3.75E-11	1.38E-17	Yes	FRMD4B
PP	20	BovineHD2000009204	31963910	1.07E-07	1.24E-10	Yes	FRMD4B
PP	20	BovineHD2000009215	31980361	1.69E-07	1.93E-10	Yes	FRMD4B
PP	20	ARS-BFGL-NGS-118998	32009781	8.33E-10	3.30E-16	Yes	FRMD4B
PP	20	BovineHD2000009226	32025240	6.49E-11	3.40E-17	Yes	FRMD4B
PP	20	BovineHD2000009234	32035193	1.63E-10	5.17E-17	Yes	FRMD4B
PP	20	BovineHD2000009251	32077537	1.08E-09	6.15E-16	Yes	FRMD4B
PP	20	BovineHD2000009459	33024411	3.74E-07	1.81E-11	Yes	FAM19A1
PP	23	BovineHD2300004717	18540309	2.39E-07		No	

Table S10. Summary of significant loci and overlaps among THI-, temperature-, relative humidity-interaction GWAS and conventional GWAS

Model	Trait	Number of significant SNPs (Conventional Main Effects) / Number of significant SNP-Trait associations (Interaction model)	Number of significant QTL regions	Number of SNPs overlapping with THI-GWAS	Percentage of overlap with THI-GWAS (%)
THI-GWAS	MY	5	1		
THI-GWAS	FP	86	4		
THI-GWAS	PP	58	8		
THI-GWAS	Total	149	13		
Temperature-GWAS	MY	6		5	83.3
Temperature-GWAS	FP	93		83	89.2
Temperature-GWAS	PP	62		56	90.3
Temperature-GWAS	Total	161		144	89.4
Humidity-GWAS	MY	6		5	83.3
Humidity-GWAS	FP	91		83	91.2
Humidity-GWAS	PP	62		56	90.3
Humidity-GWAS	Total	159		144	90.6
Conventional Main-Effect GWAS	MY	1		0	0
Conventional Main-Effect GWAS	FP	44		40	90.9
Conventional Main-Effect GWAS	PP	49		41	83.7
Conventional Main-Effect GWAS	Total	94		81	86.2
